# Susceptibility-induced distortion that varies due to motion: Correction in diffusion MR without acquiring additional data

**DOI:** 10.1016/j.neuroimage.2017.12.040

**Published:** 2018-05-01

**Authors:** Jesper L.R. Andersson, Mark S. Graham, Ivana Drobnjak, Hui Zhang, Jon Campbell

**Affiliations:** a FMRIB, Wellcome Centre for Integrative Neuroimaging, Nuffield Department of Clinical Neurosciences, University of Oxford, Oxford, United Kingdom; b Centre for Medical Image Computing & Department of Computer Science, University College London, London, United Kingdom

**Keywords:** Diffusion, Movement, Susceptibility, Registration, Dynamic

## Abstract

Because of their low bandwidth in the phase-encode (PE) direction, the susceptibility-induced off-resonance field causes distortions in echo planar imaging (EPI) images. It is therefore crucial to correct for susceptibility-induced distortions when performing diffusion studies using EPI. The susceptibility-induced field is caused by the object (head) disrupting the field and it is typically assumed that it remains constant within a framework defined by the object, (*i.e.* it follows the object as it moves in the scanner).

However, this is only approximately true. When a non-spherical object rotates around an axis other than that parallel with the magnetic flux (the *z*-axis) it changes the way it disrupts the field, leading to different distortions. Hence, if using a single field to correct for distortions there will be residual distortions in the volumes where the object orientation is substantially different to that when the field was measured.

In this paper we present a post-processing method for estimating the field as it changes with motion during the course of an experiment. It only requires a single measured field and knowledge of the orientation of the subject when that field was acquired. The volume-to-volume changes of the field as a consequence of subject movement are estimated directly from the diffusion data without the need for any additional or special acquisitions. It uses a generative model that predicts how each volume would look predicated on field change and inverts that model to yield an estimate of the field changes. It has been validated on both simulations and experimental data. The results show that we are able to track the field with high accuracy and that we are able to correct the data for the adverse effects of the changing field.

## Introduction

When an object, such as the human head, is placed in a homogeneous magnetic field it will disrupt that field and it will no longer be homogeneous. This disruption, the difference between the initial homogeneous field and the actual field, is known as a susceptibility-induced off-resonance field.

The off-resonance field presents a particular problem for echo-planar imaging because of its low bandwidth in the phase-encode direction (Jezzard and Clare, 1999). It is not unusual to have a bandwidth per pixel of ∼10 Hz for EPI images acquired without in-plane acceleration (IPAT). Typical off-resonance values for the worst affected areas (near sinuses and ear canals) are ∼150 Hz for a 3T scanner, which leads to signal displacements of up to 15 pixels.

This must be resolved and typical solutions aim at finding the off-resonance field (field map) either through direct measurement (Jezzard and Balaban, 1995, Robson et al., 1997) or by acquiring at least two EPI images that are affected differently by the off-resonance field (Andersson et al., 2003, Morgan et al., 2004, Holland et al., 2010, Irfanoglu et al., 2015). Once the field is known it is easy to correct for the first order effects of the distortions (Jezzard and Balaban, 1995, Munger et al., 2000).

As a first approximation the off-resonance field is stationary in the object (the head) framework. That is to say, if the subject moves to the right inside the scanner, the off-resonance field will also move to the right. But this is not true of any rotations around an axis non-parallel to the magnetic flux (the *z*-direction). This means that EPI images acquired with the subject in different orientations will be distorted in slightly different ways, and even after a rigid-body alignment they will not line up perfectly (Andersson et al., 2001, Ward et al., 2002, Sulikowska, 2016). To correct such a set of images with a single field map will not address the issue of differentially distorted images.

A solution to the problem would entail finding a unique field map for each EPI volume. Andersson et al. (2001) attempts to calculate the fields directly from the observed shape differences remaining in an fMRI time-series after rigid-body motion correction. Others (Boegle et al., 2010) have suggested that if a map of magnetic susceptibilities for the object is known the field can be calculated for each observed orientation of the object. Another suggestion is to use the phase information in the complex images of an fMRI time-series to get an idea of the changing field (Ooi et al., 2013, Hutton et al., 2013, Dymerska et al., 2016). Finally, Alhamud et al. (2016) has suggested acquiring a dual echo-time volumetric navigator to get a low resolution fieldmap for each volume.

However, all of these suggestions have limitations, in particular if one wants to apply them to diffusion EPI images rather than fMRI. It is not trivial to measure or deduce magnetic susceptibilities for an object, diffusion sensitisation interferes with the phase information in the images and techniques requiring non-standard acquisitions are not available on most scanners. As a consequence there is presently no widely used method to correct diffusion data for a dynamically changing susceptibility-induced field.

In this paper we present an extension of the work in Andersson et al. (2001) and Andersson and Sotiropoulos (2016). It uses the same Taylor expansion of the susceptibility field as Andersson et al. (2001), but with a generative model for diffusion data Andersson and Sotiropoulos (2015). We have validated this new method using a combination of simulations (Graham et al., 2017) and experimental data.

## Theory

### How to represent the dynamically changing field

Let us denote the susceptibility induced field in a framework that is fixed with respect to the object (the head) by ω(x) where x denotes any coordinate in the object framework. This field will depend on the orientation of the object with respect to the flux of the external field, so it is really

(1) ω(x)=ω(x:r)

which we can think of as ω(x) given the object location r within the field, where r denotes the parameters of a rigid body transform. It implies that we would have to estimate a 3D field for any point r in a six-dimensional space, which at a first glance seems intractable. However, as we outlined in the introduction, the field is only expected to change as a consequence of out-of-plane rotations. Hence, we can reduce r to [θϕ], i.e. any object rotation around the *x*- or *y*-axes. Furthermore, Sulikowska (2016) have shown that provided that *θ* and *ϕ* vary within a reasonably small range (small in this case being ±10 degrees) the field changes linearly with *θ* and *ϕ*. This means that we may be able to approximate it with a Taylor expansion around some point $r_{0}=\left[\theta_{0}\;\phi_{0}\right]$ at which we know ω(x).

(2) $$\omega \left(x:r\right)=\omega \left(x:r_{0}\right)+\text{Δ}\theta {\frac{\partial \omega }{\partial \theta }|}_{r_{0}}+\text{Δ}\phi {\frac{\partial \omega }{\partial \phi }|}_{r_{0}}+\frac{\text{Δ}\theta^{2}}{2}{\frac{\partial^{2}\omega }{\partial \theta^{2}}|}_{r_{0}}+\cdots +R_{N}$$

where N is the order of the expansion and where

(3) $${\frac{\partial \omega }{\partial \theta }|}_{r_{0}}$$

is a spatial map of the same size as ω(x) and signifies the partial derivate of the field, at each point x, with respect to *θ* at the point $\left[\theta_{0}\;\phi_{0}\right]$ (and similarly for $\partial \omega /\partial \phi {|}_{r_{0}}$, $\partial^{2}\omega /\partial \theta^{2}{|}_{r_{0}}$ etc).

For the remainder of the theory section we will limit the derivation to a first order Taylor expansion of the field where it is assumed that only rotations around the *x*- and *y*-axes affects the field. In appendix B we show how this is extended to higher order and more movement parameters.

**Fig. 1:**
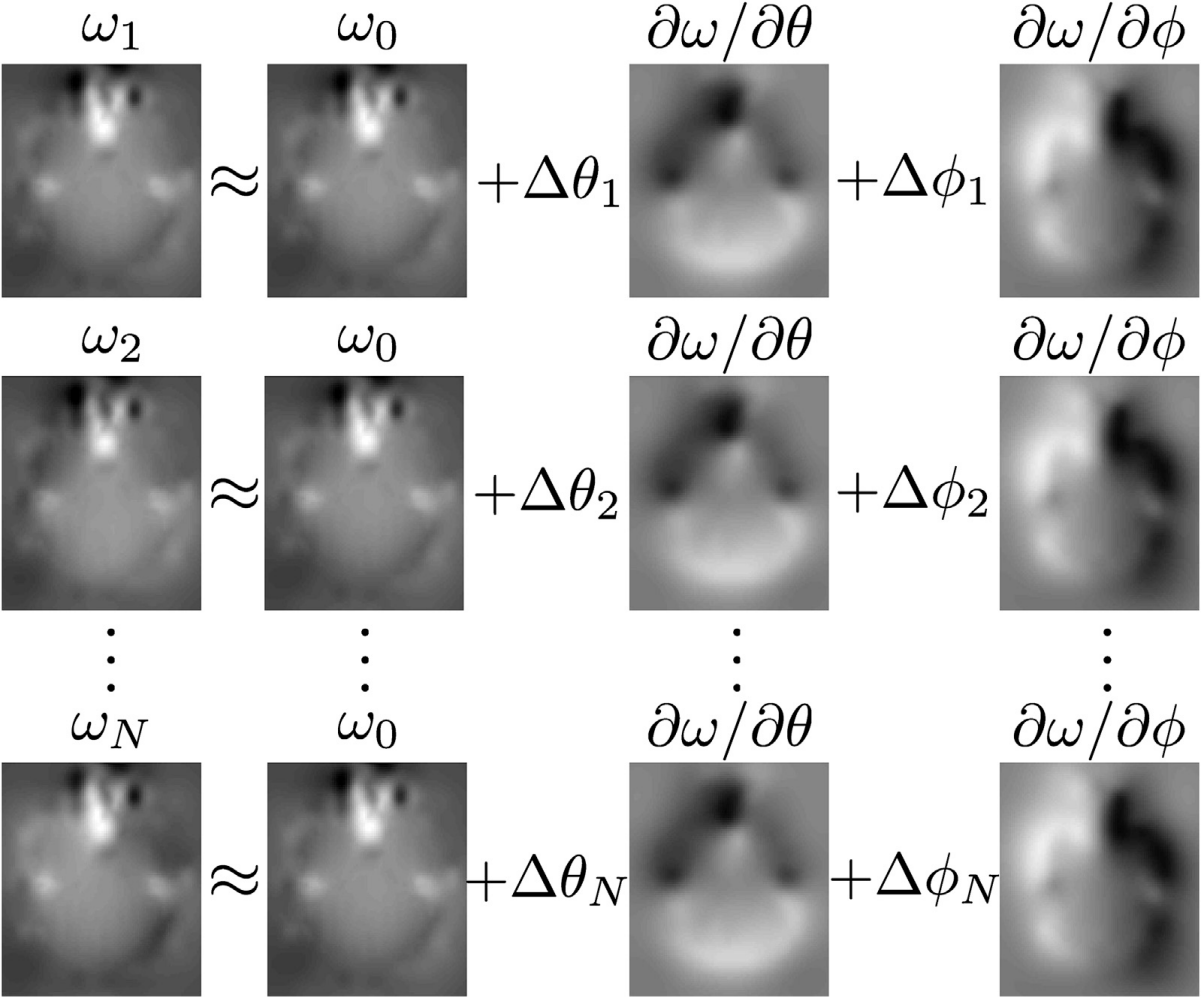
The figure is a graphical depiction of equation (2) and shows how the field $\omega_{i}$ for any volume *i* is approximated by a linear combination of a measured field $\omega_{0}$ and the derivative of that field with respect to *θ* (rotation around the *x*-axis) and *ϕ* (rotation around the *y*-axis). The weights for the derivative fields are given by the estimated movement parameters where $\text{Δ}\theta_{i}$ denotes the rotation around the *x*-axis of volume *i* relative the orientation that $\omega_{0}$ was acquired in (and correspondingly for $\text{Δ}\phi_{i}$). The maps in the figure are estimated from our simulations and the grey-scales are −40 to 100 Hz for the $w_{i}$ fields and −5 to 5 Hz/degree for the ∂ω/∂θ and ∂ω/∂ϕ fields. An intuitive description of the ∂ω/∂θ field is “How much the field changes if one nods forward (looks down) one degree”. The corresponding description for ∂ω/∂ϕ would be “How much the field changes if one tilts one's head to the right (in the direction of the dark part of the field) one degree.

Equation (2) is demonstrated in an intuitive fashion in Fig. 1.

### The model for correcting the images

We have previously described a model that we use to correct the observed diffusion images when the susceptibility- and eddy current-induced off-resonance fields as well as the subject movement have been estimated (Andersson and Sotiropoulos, 2016). It is

(4) $${\hat{s}}_{i}\left(x\right)=f_{i}\left(x^{\prime }\right)J_{x}\left(\omega ,\beta_{i},r_{i},a_{i}\right)$$

where

(5) $$x^{\prime }=R_{i}^{-1}x+d_{x}\left(\omega +e\left(\beta_{i},r_{i}\right),a_{i}\right)$$

where ${\hat{s}}_{i}$ is the corrected volume *i* and $f_{i}$ is the original volume *i*. $J_{x}$ denotes the Jacobian determinant of the transformation at location x. $R_{i}$ is a rigid-body transformation for the *i*th volume and $d_{x}$ is a voxel displacement vector for location x. *ω* and $e\left(\beta_{i},r_{i}\right)$ are scalar off-resonance fields caused by susceptibility and eddy currents respectively. $\beta_{i}$ is a vector of parameters encoding the eddy currents for the *i*th volume and $r_{i}$ is a vector of movement parameters specifying the orientation of the object in that volume. $a_{i}$ are the acquisition parameters of the *i*th volume and serves to translate the scalar off-resonance field ω+e into a vector-valued voxel-displacement value d. For many protocols $a_{i}$ would have the same value for all *i*, but in for example the HCP (Sotiropoulos et al., 2013) protocol it would take one of two values and in the dHCP (Hutter et al., 2017) protocol one of four values. A more detailed version of the spatial model used inside eddy can be found in Andersson and Sotiropoulos (2016).

For the purpose of this discourse it is useful to divide the transform into on the one hand rigid-body movement and eddy current-induced off-resonance and on the other hand susceptibility-induced off-resonance. To that end we redefine $x^{\prime }$ as

(6) $$x^{\prime }=R_{i}^{-1}x+d_{x}\left(e\left(\beta_{i},r_{i}\right),a_{i}\right)$$

so that we can rewrite equation (4) as

(7) $${\hat{s}}_{i}\left(x\right)=f_{i}\left(x^{\prime }+\omega t_{i}p_{i}^{x},\;y^{\prime }+\omega t_{i}p_{i}^{y},\;z^{\prime }\right)J_{x}\left(\omega ,\beta_{i},r_{i},a_{i}\right)$$

where *ω* is the susceptibility field in Hz, $t_{i}$ denotes the total readout time for a slice of volume *i* and $p_{i}^{x}$ denotes the *x*-component of the phase-encoding of volume *i* (and correspondingly for $p_{i}^{y}$). Typically only one of $p_{i}^{x}$ and $p_{i}^{y}$ are non-zero for a given *i* and will have a value of 1 or -1. Using the expression for *ω* from equation (2) we can rewrite equation (7) as

$${\hat{s}}_{i}\left(x\right)=f_{i}\left(x^{\prime }+\left(\omega_{0}+\text{Δ}\theta_{i}{\frac{\partial \omega }{\partial \theta }|}_{r_{0}}+\text{Δ}\phi_{i}{\frac{\partial \omega }{\partial \phi }|}_{r_{0}}\right)t_{i}p_{i}^{x},y^{\prime }+\left(\omega_{0}+\text{Δ}\theta_{i}{\frac{\partial \omega }{\partial \theta }|}_{r_{0}}+\text{Δ}\phi_{i}{\frac{\partial \omega }{\partial \phi }|}_{r_{0}}\right)t_{i}p_{i}^{y},\;z^{\prime }\right)$$

(8) $$J_{x}\left(\omega_{0}+\text{Δ}\theta_{i}{\frac{\partial \omega }{\partial \theta }|}_{r_{0}}+\text{Δ}\phi_{i}{\frac{\partial \omega }{\partial \phi }|}_{r_{0}},\beta_{i},r_{i},a_{i}\right)$$

where $\text{Δ}\theta_{i}$ and $\text{Δ}\phi_{i}$ are the rotations around the *x*- and *y*-axes respectively of volume *i* compared to the reference $r_{0}$ orientation.

### Adding Gaussian Process predictions

For the following we will assume the existence of an independent prediction ${\overset{\text{˘}}{s}}_{i}$ of how the corrected image ${\hat{s}}_{i}$ from equation (8) should look. This is obtained using a Gaussian Process (GP) and is a linear combination of all the diffusion weighted images (DWIs) corrected for the current estimates of distortions and movements. It has been described in detail in Andersson and Sotiropoulos, 2015, Andersson and Sotiropoulos, 2016. Assuming that ${\overset{\text{˘}}{s}}_{i}\approx {\hat{s}}_{i}$ we can write

$${\overset{\text{˘}}{s}}_{i}\left(x\right)=f_{i}\left(x^{\prime }+\left(\omega_{0}+\text{Δ}\theta_{i}{\frac{\partial \omega }{\partial \theta }|}_{r_{0}}+\text{Δ}\phi_{i}{\frac{\partial \omega }{\partial \phi }|}_{r_{0}}\right)t_{i}p_{i}^{x},y^{\prime }+\left(\omega_{0}+\text{Δ}\theta_{i}{\frac{\partial \omega }{\partial \theta }|}_{r_{0}}+\text{Δ}\phi_{i}{\frac{\partial \omega }{\partial \phi }|}_{r_{0}}\right)t_{i}p_{i}^{y},\;z^{\prime }\right)$$

(9) $$J_{x}\left(\omega_{0}+\text{Δ}\theta_{i}{\frac{\partial \omega }{\partial \theta }|}_{r_{0}}+\text{Δ}\phi_{i}{\frac{\partial \omega }{\partial \phi }|}_{r_{0}},\beta_{i},r_{i},a_{i}\right)+\varepsilon$$

where ε is the “error” or the deviation between ${\overset{\text{˘}}{s}}_{i}$ and ${\hat{s}}_{i}$. For the case where $\partial \omega /\partial \theta {|}_{r_{0}}$, $\partial \omega /\partial \phi {|}_{r_{0}}$ etc are unknown (or not fully known, like in the early stages of the iterative estimation) that error signal will contain information about $\partial \omega /\partial \theta {|}_{r_{0}}$, $\partial \omega /\partial \phi {|}_{r_{0}}$ etc.

### Estimating the derivative fields

The formal derivation of the equations for estimating the field is in appendix A. Below we present an intuitive explanation of the method.

#### Intuitive explanation of our proposed method

Our explanation depends on two notions. The first is that there is a small number of “maps” that capture the variability of the off-resonance field over the course of an experiment. The second notion is that the residual variance, *i.e.* the difference between observations and predictions, contains enough information to allow us to estimate these “maps”.

These maps represent one anatomical location per voxel, as defined by the reference (first) volume, and specifies the linear change with respect to the parameter in question (pitch or roll) at that location. Specifically is it the slope of a linear relationship between the strength of the off-resonance field and orientation parameter (pitch or roll) in units of Hz per degree. Formally these maps are maps of the partial derivative of the off-resonance field with respect to pitch and roll.

**Fig. 2:**
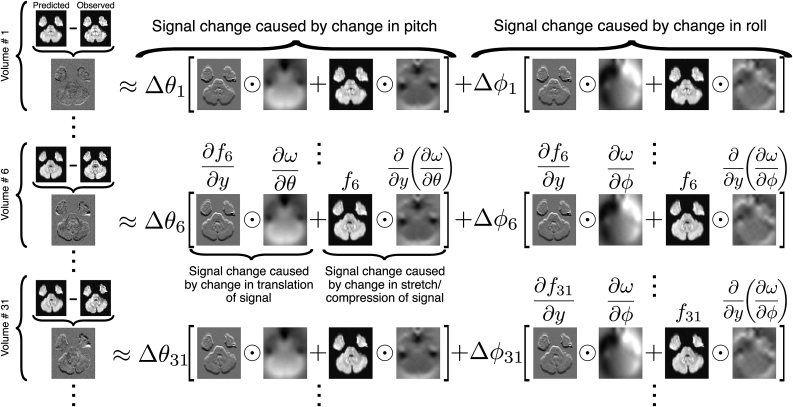
This figure illustrates the forward model that explains the difference between observations and predictions as the result of a changing off-resonance field. It demonstrates how the total difference can be described as the sum of the signal changes caused by a change in pitch and the signal changes caused by a change in roll. The signal changes can be further subdivided into changes in translation of the signal along the PE-direction (*i.e.* how the sampling point in the predicted image changes) and changes in Jacobian modulation (intensity changes caused by stretching/compression). The choice of volumes 1, 6 and 31 for demonstration is arbitrary and the model of course encompasses all volumes (both b=0 and DWI volumes). The symbol ⊙ was used to denote Hadamard (or elementwise) product.

Fig. 2 illustrates the forward model that is used. It shows how the observed differences are explained in terms of off-resonance changes caused by changes in pitch and changes in roll. Furthermore it demonstrates how these can be subdivided into changes in deflection of the signal and changes in stretching/compression of the signal, both along the PE-direction (in the figure the *y*-direction is the PE-direction).

To get an intuition for this consider, for example, the part that is given by $\text{Δ}\theta_{6}\left(\partial f_{6}/\partial y\right)⊙\left(\partial \omega /\partial \theta \right)$ halfway down on Fig. 2. The map ∂ω/∂θ denotes the rate of change of the field with respect to pitch in units of Hz/degree, so when multiplying this with $\text{Δ}\theta_{6}$ (degrees) we get a map with the change in field (in Hz) as a consequence of pitch change for volume # 6.

In this forward model it is assumed that the derivative fields (∂ω/∂θ and ∂ω/∂ϕ) are known, but these are of course the maps we are interested in estimating. So, the next step is to describe a model to estimate the unknown entities ∂ω/∂θ and ∂ω/∂ϕ.

**Fig. 3:**
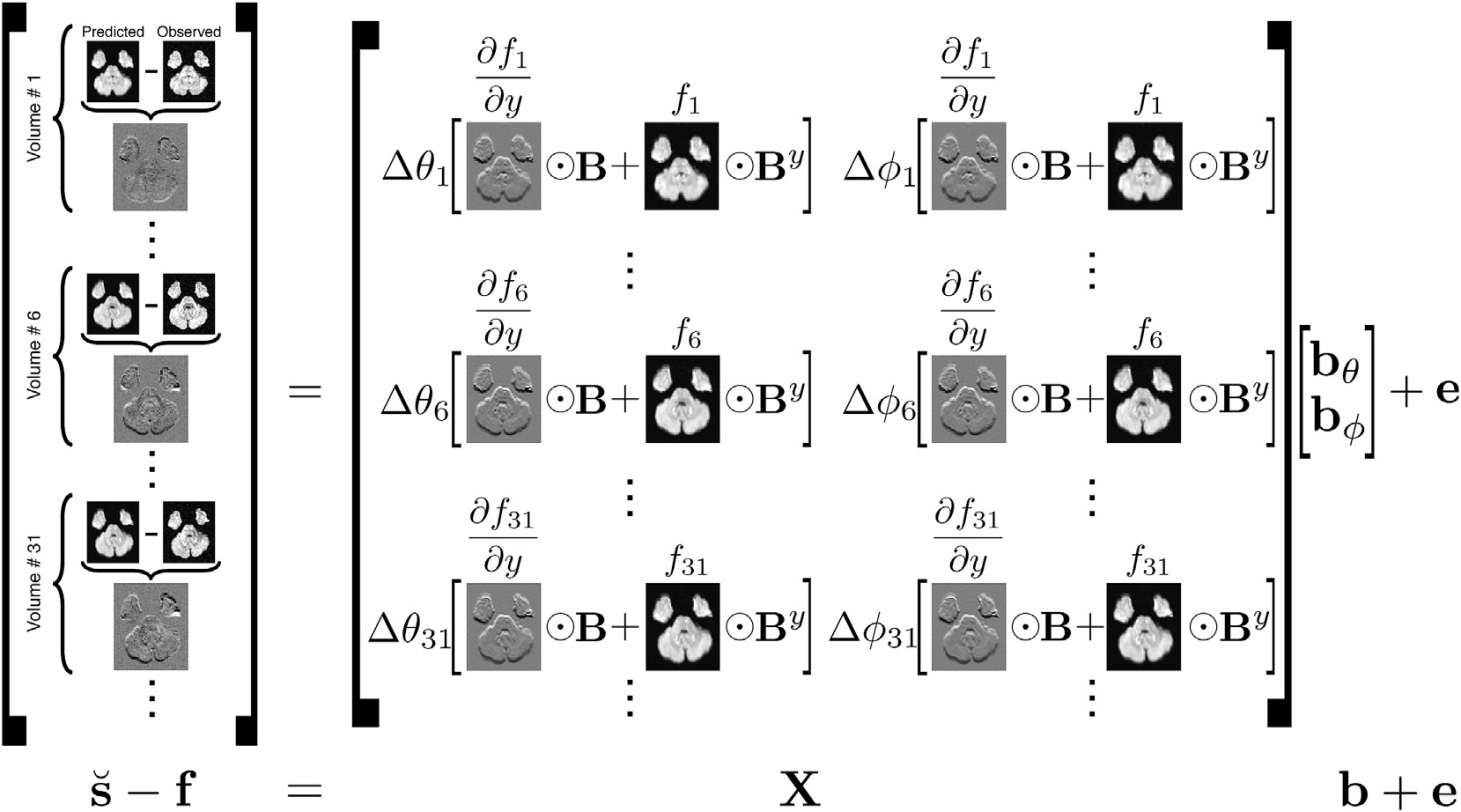
This figure shows the matrix-vector equation $\overset{\text{˘}}{s}-f=Xb+e$ that is solved for $\hat{b}$ in a least squares sense. The matrix X implements the forward model described in Fig. 2, but with a spatial basis set B in lieu of the (unknown) derivative fields. The vector $b={\left[b_{\theta }^{\text{T}}\;b_{\phi }^{\text{T}}\right]}^{\text{T}}$ contains the weights for the fields ∂ω/∂θ and ∂ω/∂ϕ such that $B{\hat{b}}_{\theta }$ is an estimate of ∂ω/∂θ. $B^{y}$ denotes a matrix that is organised in the same way as B, but where the columns consists of splines that have been differentiated in the *y*-direction (PE-direction).

Fig. 3 illustrates the forward model from 2 as a large system of linear equations described in matrix vector notation. In this figure the unknown fields ∂ω/∂θ and ∂ω/∂ϕ are now represented as $Bb_{\theta }$ and $Bb_{\phi }$ respectively. The matrix B contains a B-spline basis set that covers the entire 3D space of a data volume. The vector b consists of two “sub-vectors”, $b_{\theta }$ and $b_{\phi }$, that contain the weights for the B-spline basis such that the resulting weighted sums are the estimated fields.

In the formulation in Fig. 3 $\overset{\text{˘}}{s}-f$ is a very tall ($Nn_{x}n_{y}n_{z}\times 1$) column vector, that is modeled as the product of a matrix X calculated from known entities and an unknown vector b. This is simply an over determined system of linear equations that can be solved in a least squares sense. However, the forward model described in Fig. 2 is a linearisation of a problem that is fundamentally non-linear with respect to the unknowns (b). Therefore solving the linear system in Fig. 3 represents the linearisation in a Gauss-Newton scheme and has to be repeated iteratively, each time updating b such that

(10) $$b^{\left(k+1\right)}=b^{\left(k\right)}+{\left(X^{\text{T}}X\right)}^{-1}X^{\text{T}}\left(\overset{\text{˘}}{s}-f\right)$$

where X and f both depend on $b^{\left(k\right)}$.

In appendix A we provide the formal derivation of the Gauss-Newton step in equation (10) and that is the proper reference for the proposed method. The calculation of $X^{\text{T}}X$ in equation (10) above is potentially very computationally expensive. The reason for that, and how to reduce some of that expense, is explained in appendix C.

### Combining the model with estimation of eddy currents

There is an extra caveat with the method outlined in the previous sections when there are also eddy current-induced distortions, as explained in this section.

Looking at the derivative fields in Fig. 1 it is clear that there are strong linear components in both ∂ω/∂θ and ∂ω/∂ϕ. This means that if one were to model the change of the field for those images as just a linear gradient (in the *y*-direction for ∂ω/∂θ and in the *x*-direction for ∂ω/∂ϕ) it would already explain a fair proportion of the variance seen in the data (this is the idea behind some attempts at real-time shimming (Ward et al., 2002, Alhamud et al., 2016)). And for diffusion weighted images we estimate a low order eddy current-induced field for each volume, hence this will include also the low (spatial) order parts of the susceptibility-by-movement interaction.

In itself it is not necessarily a problem that the EC-correction subsumes the low frequency component of the derivative field. One would expect the estimated susceptibility derivative field to be the high frequency component of the true derivative field, and that the superposition of the change of the susceptibility and the estimated EC field would still be correct.

However, the b=0 volumes do not suffer from EC-induced distortions, so none is modeled. That means that the derivative field one needs for the b=0 volumes is the “full field”, *i.e.* both the low and high spatial frequencies. That leads to problems if one wants to model a joint derivative field for the b=0 and the diffusion weighted images alike.

The option to model separate fields for b=0 and diffusion volumes would be suboptimal for several reasons. It would increase computational effort, but above all it would negatively affect the precision of the estimated fields and potentially introduce shape differences between the corrected b=0 and diffusion volumes.

We have therefore opted for a scheme where we attempt to estimate the “full field” (low and high spatial frequencies) for b=0 and diffusion weighted volumes alike. It is done through a two step process where an initial set of EC parameters is estimated while limiting the estimates by a linear causal model of the diffusion weighting (referred to as a “linear second level model” in Andersson and Sotiropoulos (2016)). This limits the risk that the EC-model attempts to “explain” the shape differences that has been caused by the susceptibility field varying with subject orientation. However, this can potentially lead to suboptimal results because i) the linear second level model may be too strict and give slightly suboptimal results (Andersson and Sotiropoulos, 2016) and ii) the EC-model can still “explain” some susceptibility shape differences. For these two reasons, a second step is employed where iterations for estimating the susceptibility derivative fields (*i.e.* equation (A6)) are interleaved with iterations for estimating EC-parameters (without a second level model), subject movement and outliers (Andersson and Sotiropoulos, 2016, Andersson et al., 2016, Andersson et al., 2017). A schematic of the algorithm is as follows:

**Figure:**
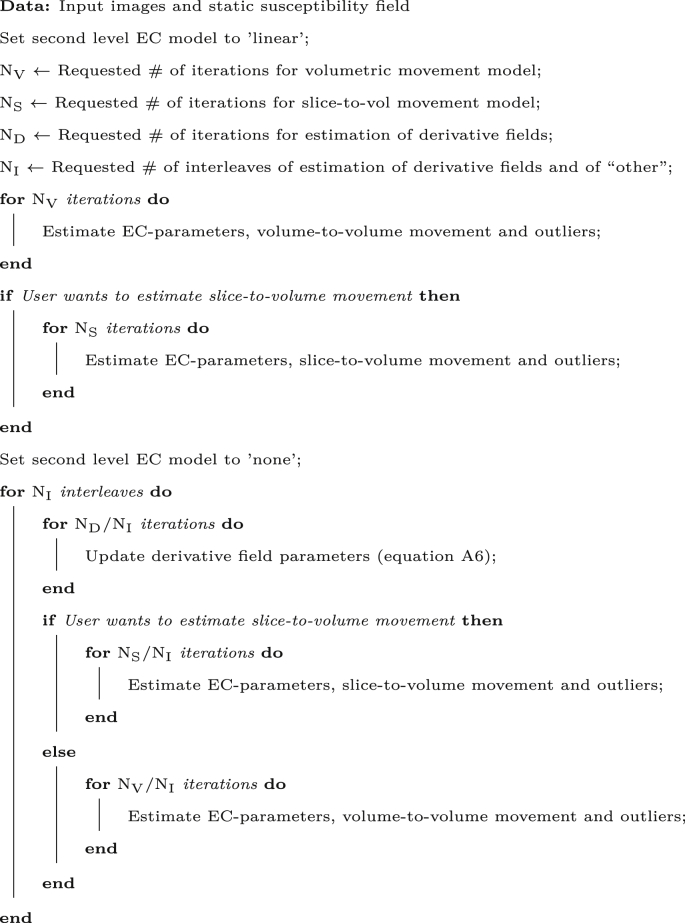

### Combining the model with estimation of intra-volume movement

We expect this method to be useful primarily for data/populations where there is a relatively high degree of movement. Therefore it is likely that the data will also be marred by intra-volume movement, *i.e.* there will be volumes where the subject has moved during the acquisition of the slices/MB-groups that constitute the volume. This results in a stack of slices that may be translated and/or rotated relative each other and may not even be parallel. It is important to ensure that the two corrections (intra-volume and susceptibility-by-movement) work seamlessly together.

As can be seen in Fig. 2, Fig. 3 our model for estimating the derivative fields operates with a volume as the unit. When the algorithm (see algorithm 1 above) reaches the stage where it estimates the derivative fields, one set of intra-volume movement parameters have already been estimated and used to correct the data that goes into the estimation of the derivative fields. Hence, the problem is *not* primarily that volumes contaminated by intra-volume movement “confuse” the estimation of the fields. The problem is that volumes where the subject has performed a large movement (in particular if that includes a pitch and/or roll) have not been acquired under the influence of a well defined field.

We have solved that by examining the (slice-wise) movement parameters for each volume after the initial step (prior to the estimation of the derivative fields) to identify all volumes with an intra-volume standard deviation of Δθ and/or Δϕ greater than one degree. These volumes are considered “poorly defined” with respect to orientation, and they are excluded from the estimation of the derivative fields. However, it should be noted that they are included in the movement-eddy-current-outlier estimations that are interleaved with the derivative field estimation. That way these parameters can still be updated for those volumes in the light of the derivative fields. It should also be noted that when building the field for use in correcting a given volume, we use the intra-volume parameters (provided a slice-to-volume model was used in the estimation) so that the Δθ and Δϕ that are actually used in equation (2) vary from slice-to-slice/group-to-group.

In the simulations described below the method was tested with both a volumetric and a slice-to-volume movement model. For the experimental data with large subject movements it was inevitable that there would also be large intra-volume movement and only the slice-to-volume model was used to analyse those.

## Material and methods

### Implementation

The method described in the present paper has been implemented in C++ as part of the eddy tool (Andersson and Sotiropoulos, 2016 and http://fsl.fmrib.ox.ac.uk/fsl/fslwiki/EDDY) in FSL (see Smith et al. (2004) and http://fsl.fmrib.ox.ac.uk/fsl/fslwiki/). Some of the steps of the algorithm, in particular the calculation of the derivatives and the spline-interpolation on an irregular grid, are very computationally intense. They have therefore been parallelised for NVidia GPUs using CUDA (Farber, 2011). All the results in the present paper have been generated using the GPU version of the software.

The current version of eddy will estimate parameters for eddy current-induced off-resonance fields and subject movement (see equations (4), (5)) and Andersson and Sotiropoulos (2016)). In addition it will also detect and replace movement induced signal dropout (Andersson et al., 2016) and correct intra-volume movement (Andersson et al., 2017). The work presented here has extended that to also include susceptibility-by-movement effects.

The new addition has increased the execution time for eddy. Not so much because of the actual estimation of the fields, but more for the additional iterations for estimation of eddy currents and movement that are interleaved with the field estimation. Hence, it effectively doubles the execution time compared to running eddy without the susceptibility-by-movement correction.

### Simulations

#### Data

The simulations that we use are similar to those described in Graham et al. (2017) where the authors characterise the problem that the method in the present paper is attempting to solve. In brief, the data was simulated using POSSUM, a highly realistic MRI simulator (Drobnjak et al., 2006) that has been extended to diffusion imaging (Graham et al., 2016). It contains an input object (head) that has been segmented into air and tissue and from which a susceptibility induced-field can be calculated for any orientation relative to the magnetic flux (Jenkinson et al., 2004).

Using POSSUM, a diffusion data set was simulated with 60 b=1000 volumes and five interspersed b=0 volumes, where the diffusion directions were optimised on the whole sphere and were identical to those used for the FMRIB standard diffusion protocol. The simulated acquisitions were single-band with a 72×96×55 matrix, an interleaved slice ordering and an isotropic voxel size of 2.5 mm. The total readout time for a slice was 77.4 ms, corresponding to a bandwidth of 12.9 Hz/pixel in the PE-direction. The susceptibility field was calculated for a 3T static field and the eddy currents (when applicable) were commensurate with a Stejskal-Tanner diffusion encoding.

Various other options were varied according to a 2×2×2×2×2 factorial. The factors that were varied were

**Movement type:** Volumetric or continuous movement. We expect this method to be of particular use for subjects that move more than average. Therefore we simulated data both with the movement injected “per volume” (a commonly used model) and with movement continuous in time (a more realistic model (Andersson et al., 2017)). The latter will result in a subset of “broken” volumes with movement between consecutive slices such that when stacked up they no longer represent anatomically faithful volumes.

**Movement magnitude:** Normal or large movement. The “large” movement trace was taken from a volunteer moving according to an agreed pattern including pitch and roll (rotation around the *x*- and *y*-directions). The “normal” movement was created from the same trace, but with all parameters divided by three.

**Eddy current distortions:** With or without eddy current-induced distortions.

**Phase-encode direction:** With phase-encode direction A→P or P→A.

**SNR:** With an SNR of 20 or 40. The SNR is defined from the b=0 volumes as the average intra-cerebral signal divided by the noise.

Ten distinct simulations with unique noise were performed for each bin in this hyper-cube, resulting in a total of 320 data sets each consisting of 65 vol.

In addition to the simulations described above we also simulated data with exactly the same protocol but without any movement or off-resonance fields. These “ground truth” data sets were simulated without noise, or with added noise to yield b=0 SNR of 20 and 40.

#### Analysis

If nothing else is specified the analyses below was performed using a first order Taylor expansion around *x*- and *y*-rotation, a spline knot-spacing of 10 mm, 20 iterations and a regularisation *λ* of 10 (see equation (A13)). The static field was estimated from a pair of A→P and P→A b0-volumes (using the FSL-tool topup, Andersson et al. (2003)) from the simulations. These were the first volumes from their respective data sets, so before any movement was injected. Movement was estimated either using a volumetric model or with a slice-to-volume model with 17 degrees of freedom per volume.

##### Estimating the “true” derivative fields

The susceptibility fields used for the simulations were generated in a framework that was fixed with respect to the object (head). Therefore these were all reoriented to the space of the first volume (the reference space for eddy) using the known movement parameters, after which the phase was fitted to a general linear model consisting of a constant and the (known) rotations around the *x*- and *y*-axes (Δθ and Δϕ respectively). The resulting parameter maps for Δθ and Δϕ are considered as the “true” ∂ω/∂θ and ∂ω/∂ϕ fields.

##### Checking the assumptions of the Taylor expansion

One of the assumptions of the model is that a first order Taylor expansion is sufficient to track the changes of the field with head orientation. This was tested on the simulated data by generating $R^{2}$-maps for a first order model. First the “true” first order derivative fields were calculated, as described above, after which the fraction of variance explained by these was calculated.

##### Comparing estimated and true derivative fields

The “true” derivative fields were estimated as described above and compared to the ∂ω/∂θ and ∂ω/∂ϕ maps estimated using the method described in the present paper.

##### Comparing estimated and true off-resonance fields

For each volume we had full knowledge of the actual off-resonance field that had affected that acquisition. We were able to compare that, on a volume-by-volume basis, to the estimated field. The estimated field was calculated in two ways, as

• The sum of the static susceptibility field estimated with topup and the eddy current-induced field estimated by eddy.
• The sum of the static susceptibility field (topup), the eddy current-induced field and the change in the susceptibility induced-field caused by the change of orientation of the head. The two latter estimated by eddy.

This analysis was limited to the data simulated with volumetric movement because the slice-to-volume case does not have fields that are defined for entire volumes.

##### Comparing corrected and true images

After correcting the simulated data for movement and distortions it was compared to the “ground truth” data simulated without any movement or off-resonance effects. This was done by calculating the correlation between paired (with the same diffusion encoding) volumes. We compared the results when excluding or including susceptibility-by-movement effects in the correction.

The effects of the diffusion encoding direction (being fixed in the scanner framework) changing in the subject framework as the subject rotates its head (Leemans and Jones, 2009) were *not* included in any of our simulations. Hence, the volume pairs should have identical contrast.

### Human data

A healthy and experienced volunteer was scanned three times in the same session, twice with the FMRIB standard diffusion protocol and once with an augmented protocol. The standard protocol consists of 60 unique directions with a *b*-value of 1500 and five interspersed b=0 volumes. The augmented protocol had the same 60 directions with a *b*-value of 1500, but instead of five b=0 volumes it had a total of 61 b0-volumes such that the full sequence was “b0, b0, DWI, b0, DWI, b0 … b0, DWI”. Each of these scans was preceded by the acquisition of two b=0 volumes with identical scanning parameters except for the phase-encode direction being reversed. For the first scan the subject was instructed to lie as still as possible. For the second scan he was instructed to make a set of movements (rotations around the *x*- and *y*-axis), where after each movement the new position was held for ∼10 volumes. For the third scan the instruction was to try and mimic the movement from the second scan, but this time hold each new position for ∼20 volumes. Note that, similarly to the simulations with intra-volume movement injected, this means that the human data contained volumes contaminated by intra-volume movement.

The data was acquired on a Siemens Magnetom Prisma system with a 32-channel receive head coil and monopolar Stejskal-Tanner gradients were used for the diffusion encoding. The resolution was 1.5 mm isotropic with 84 slices of a 150×150 matrix. A multi-band factor of 4 was used, no in-plane acceleration, phase-encoding A→P and a 6/8 partial *k*-space. The TR was 3.35 s, the TE was 88.8 ms and the total readout-time (time between centre of first and last echo) was 107 ms, yielding a bandwidth per voxel of 9.32 Hz.

#### Analysis

We performed several analyses of the data to explore different aspects of the method. If nothing else is stated all estimations/corrections below were performed with a slice-to-volume movement model with 17 degrees of freedom per volume, eddy current correction and detection and replacement of outliers. The susceptibility-by-movement estimation was performed using a knot-spacing of 10 mm, a regularisation *λ* of 10 and 20 iterations in 5 interleaves with movement, EC and outlier estimation as outlined in algorithm 1.

##### Test-retest reproducibility of the estimated derivative fields

By excising relevant b0-volumes from the third scan we mimicked the data from the second scan. Thus we had two corresponding, but independent, data sets with similar levels of movement from which to estimate the ∂ω/∂θ and the ∂ω/∂ϕ fields. In order to ensure that we performed the Taylor expansion around the same point in orientation space we used the static field estimated from scan two for both scans. We also replaced the first b=0 in the third scan by the first b=0 from the second scan, thus ensuring the same reference space for both analyses.

##### Ability to estimate derivative fields from diffusion data

It is potentially more difficult to estimate the derivative fields from diffusion data than from data with inherently similar contrast. The differing contrast/information content in the different diffusion weighted volumes is one complication, and there is also the difficulty of estimating it in the presence of volume specific eddy current-induced distortions. To investigate that, the third scan was sub-sampled into two data sets, one where the additional b=0 volumes had been stripped away to yield a data set consisting of 60 diffusion weighted volumes and 5 interspersed b=0 volumes as in the standard protocol. The other sample consisted only of the 61 b=0 volumes. For this analysis the static field was estimated from the first b=0 volume from the third scan and the immediately preceding b=0 scan with reversed PE-direction. The two samples have the same initial volume, so the reference space was the same. The estimation from b=0 data was performed in the same way as for the diffusion weighted data, except that eddy currents were not estimated.

##### Ability to correct the data for susceptibility-by-movement effects

For this analysis the images acquired in the first scan were considered as “ground truth”. Both visual inspection and the estimated movement parameters confirmed that there were very little movement and no obvious movement related dropout. It was corrected using a volume-to-volume movement model, eddy current correction and detection and replacement of outliers. Data set two and three (the latter after stripping of extraneous b=0 volumes) were used as “test” data sets. They were corrected as described above, once with and once without susceptibility-by-movement estimation. The fields estimated from the reversed PE-direction data were used as the static fields. After within-scan correction a rigid-body motion transform was calculated for scans 2 and 3 relative to scan 1, and the former data sets were resampled into the space of scan 1 using spline interpolation. This entails an additional interpolation, but that should not matter for the comparison between results with and without susceptibility-by-movement estimation.

Unlike the simulations, these data will be affected by the diffusion encoding direction rotating in the subject framework when the subject rotates its head. However, that should only lead to a slightly lower correlation for the volumes with the greatest rotation and will not bias the comparison between results with and without susceptibility-by-movement.

## Results

### Simulations

Looking at movies of the simulated data (Fig. S1 in the supplementary material) after correcting for eddy currents, subject movement and a static susceptibility field it was clear that in the areas with a strong off-resonance field from susceptibility it was not sufficient to correct with a single field. The susceptibility-by-movement effects were obvious as apparent shape changes between volumes. It was also clear that these were very well corrected by the susceptibility-by-movement model.

#### Checking the assumptions of the Taylor expansion

**Fig. 4:**
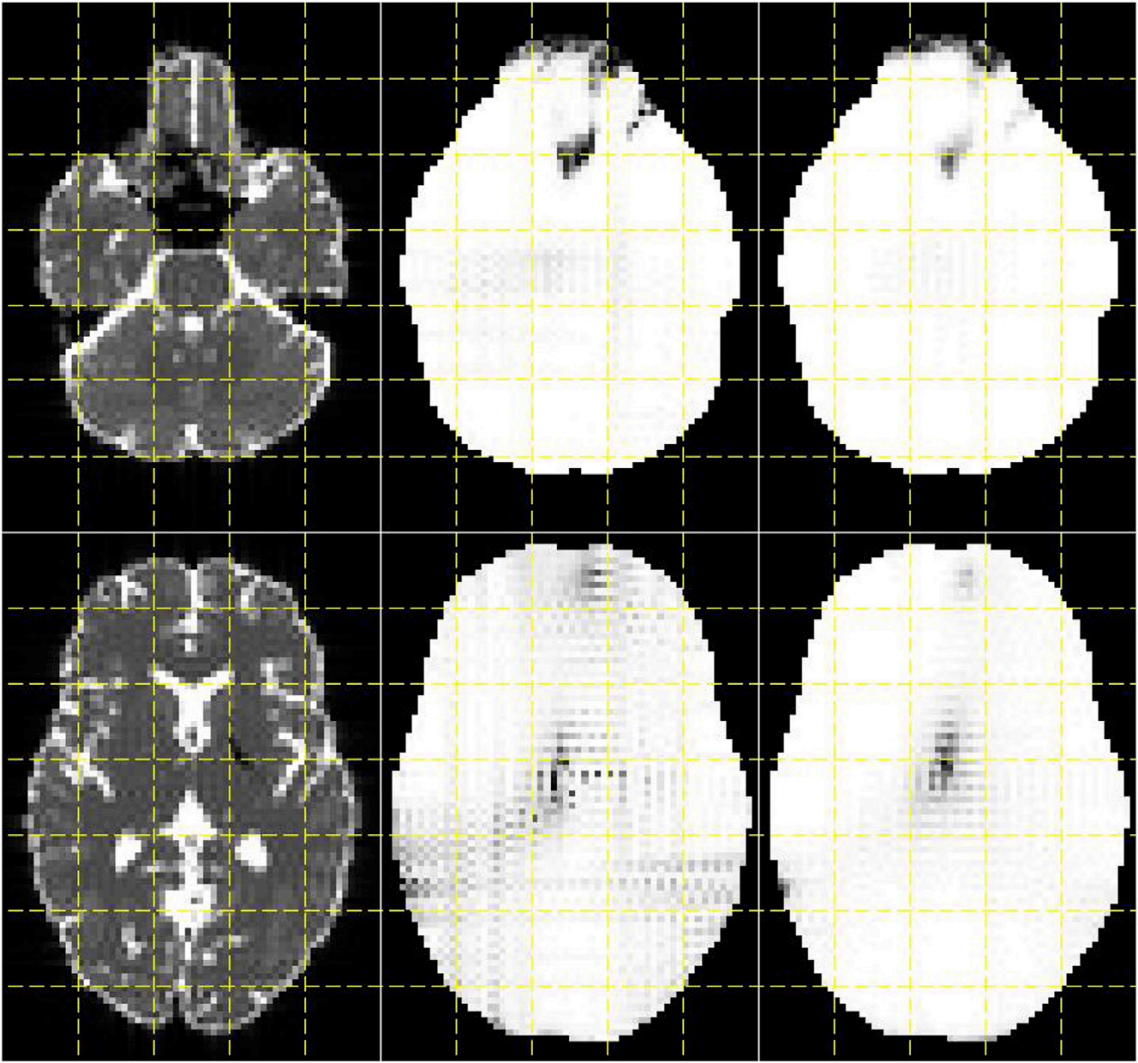
This figure shows the $R^{2}$-maps that demonstrate the proportion of true variance in the simulated off-resonance fields explained by a first order model. The left column shows b=0 images for anatomical guidance. The middle and rightmost columns show the $R^{2}$-maps for the “normal” and “large” movement simulations respectively. The grey-scale goes from 0 (black) to 1 (white).

Fig. 4 shows the $R^{2}$-maps for two different levels of the brain. It can be seen that the maps are close to 1 for most of the intra-cerebral voxels. In fact they are greater than 0.99 for most of the brain at these levels. The regions that are less than 0.99 coincide with areas where it was problematic to estimate the “true” field due to phase wrapping. Together with the observation that there are fewer voxels with an $R^{2}$ less than 0.99 for the large movement case makes us conclude that a first order model is sufficient for the simulations. If those areas had truly reflected a need for a higher order expansion one would have expected more voxels with $R^{2}$ less than 0.99 for the large compared to the normal movement case.

#### Comparing estimated and true derivative fields

**Fig. 5:**
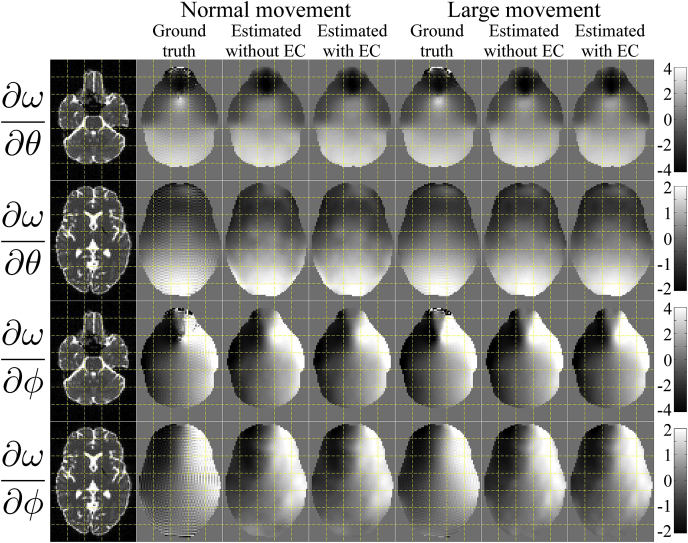
This figure shows the true and estimated maps of ∂ω/∂θ and ∂ω/∂ϕ (top two and bottom two rows respectively) when using simulations with volumetric movement, phase-encoding A→P and an SNR of 40. The first column shows the true b=0 images for anatomical guidance. Columns 2–4 show the results for the “normal” movement case. Column 2 shows the “truth”, column 3 the estimated fields for the “No eddy currents” case and column 4 the estimated fields when they were jointly estimated with eddy current-induced fields. Correspondingly columns 5–7 show the results for the “large” movement case, where column 5 is the “truth” and columns 6 and 7 shows the estimated fields in the absence and presence of eddy current-induced fields respectively. The units of the colorbars are Hz/degree.

Fig. 5 shows estimated versus “true” derivative fields for some of the simulations. The particular simulations used for Fig. 5 used a volumetric movement model, phase-encoding in the A→P direction and an SNR of 40. It can be seen that the estimated derivative fields capture the features of the true fields very well for both “normal” and “large” movements and regardless of the presence of eddy current-induced distortions.

The correlations between “true” and estimated derivative fields are tabulated for all the different simulations in Tables S1 and S2 in the supplementary material S. Fom the results in Fig. 5 and Tables S1 and S2 we conclude that

• We are able to accurately estimate the rate of change of the field from the diffusion data.
• That ability is largely independent of PE-direction, the absence or presence of eddy-currents, the absence or presence of intra-volume movement artefacts and of SNR in the range 20–40.
• The one things that appears to have an appreciable effect on that ability is the magnitude of movement. We believe that is partly explained by a poorer “estimate of truth” (can be seen as ringing artefacts in Fig. 5) for the normal movement case. But also that it reflects less information about the susceptibility-by-movement in data with less movement.

#### Comparing estimated and true off-resonance fields

**Fig. 6:**
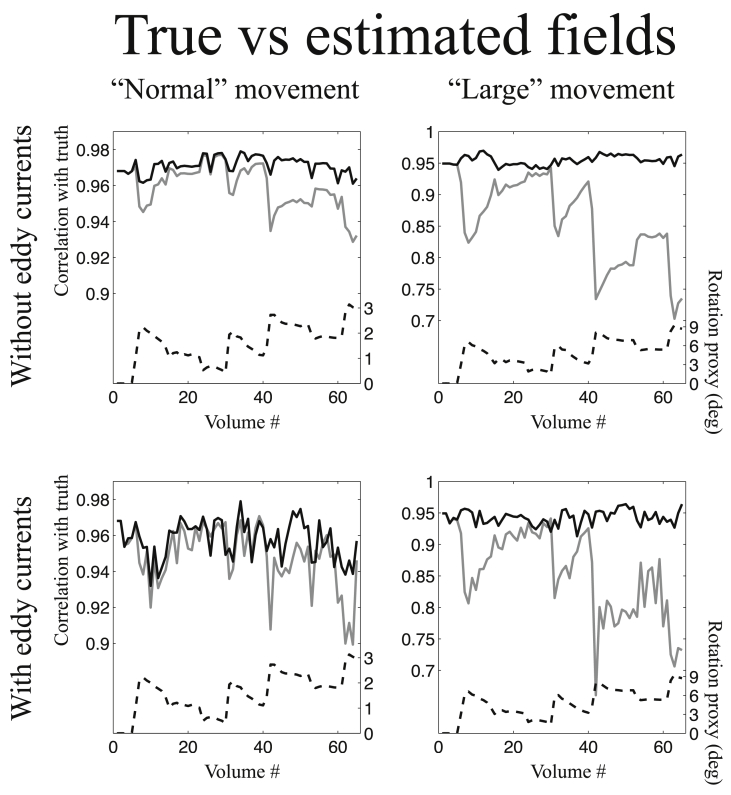
This figure shows the correlation between true and estimated off-resonance fields for all volumes of the simulated data. The data used for this figure was simulated such that the phase encode direction was A→P and the SNR was 40. The solid black and solid grey lines represent the correlation between true and estimated off-resonance fields for the method in the present paper and the method assuming a constant susceptibility field respectively. The scale for both these curves is found on the left-hand of the graphs. The dashed black line shows a “proxy” for the rotation relevant to susceptibility field. It was calculated as $\sqrt{R_{x}^{2}+R_{y}^{2}}$ where $R_{x}$ and $R_{y}$ denote rotation around the *x*- and *y*-axes respectively. The scale for that curve is found on the right hand side of the graphs. The graphs in the left column show the results for “normal” subject movement and the right column for “large” movement. The top row shows the situation when no eddy current distortions were simulated and no attempt was made to estimate eddy currents. The bottom row shows the situation when eddy currents were included in the simulations and eddy currents and susceptibility-by-movement were jointly estimated by eddy.

The results from the comparison of estimated and true off-resonance fields are shown in Fig. 6. It shows the simulations for phase-encode direction A→P and an SNR of 40. The results for phase-encode direction P→A and SNR 20 are sufficiently similar that we have chosen to not include those results. As expected it shows that the advantage of our method is greater for “large” than for “normal” movement, though there is a clear advantage also for “normal” movement. The loss of accuracy of the estimated fields when not taking susceptibility-by-movement into account (grey lines) is strongly related to a “rotation proxy” (dashed black line) that we calculated as a composite measure of the *x*- and *y*-rotations (see legend to Fig. 6). It can also be seen that the accuracy of the estimated fields were poorer when the simulated data contained eddy current-induced distortions. However, the relative increased accuracy offered by the method in the present paper remained the same.

#### Comparing corrected and true images

**Fig. 7:**
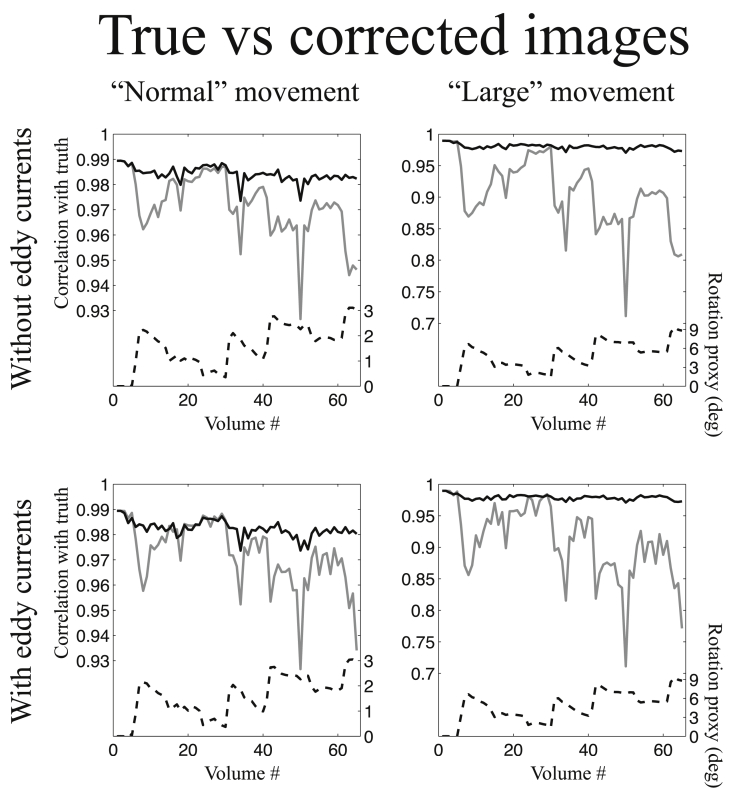
This figure shows the correlation between true and corrected images for all volumes of the simulated data. The data used for this figure was simulated such that the phase encode direction was A→P, the SNR was 40 and all movement was “inter-volume”, *i.e.* any movement was assumed to occur between acquisition of consecutive volumes. Correspondingly the analysis used a volumetric movement model. The solid black and solid grey lines represent the correlation between true and corrected images for the method in the present paper and the method assuming a constant susceptibility field respectively. See the legend for Fig. 6 for more details.

**Fig. 8:**
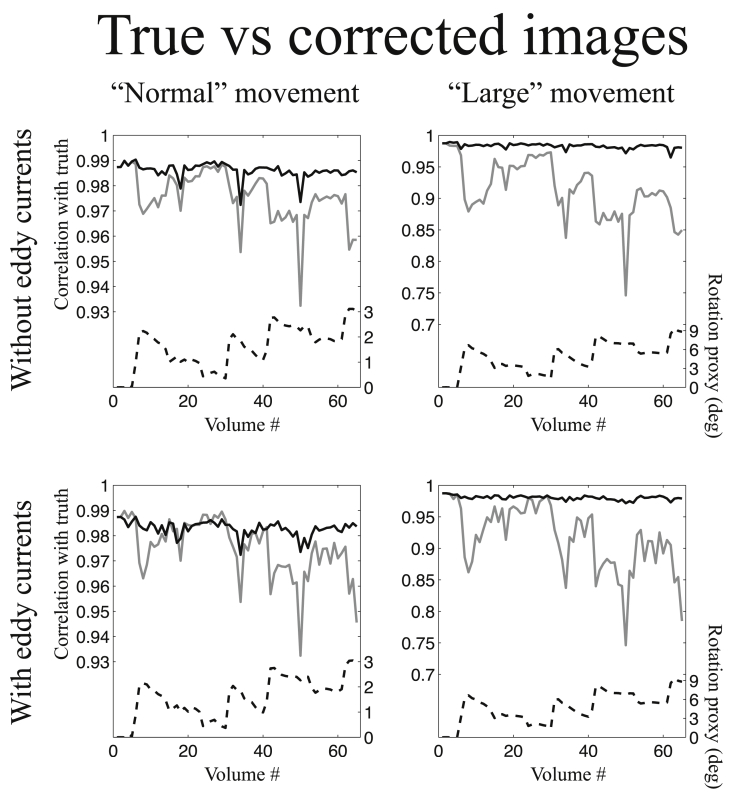
This figure shows the correlation between true and corrected images for all volumes of the simulated data. The data used for this figure was simulated such that the phase encode direction was A→P, the SNR was 40 and movement was continuous, *i.e.* volumes occurring during periods of rapid movement were corrupted by intra-volume movement. Correspondingly the analysis used a slice-to-volume movement model. The solid black and solid grey lines represent the correlation between true and corrected images for the method in the present paper and the method assuming a constant susceptibility field respectively. See the legend for Fig. 6 for more details.

Fig. 7, Fig. 8 show the correlation between true and corrected images for a volumetric and slice-to-volume movement model respectively. The correlation was calculated for a single slice corresponding to the most basal slice shown in Fig. 4, Fig. 5, *i.e.* a slice that is appreciably affected by susceptibility-by-movement effects. These (Fig. 7, Fig. 8) correspond to the simulations with phase-encoding in the A→P direction and an SNR of 40. Similar figures for both PE-directions, both SNR levels (20 and 40) and for slices at different levels (in the inferior-superior directions) of the brain are shown in Figs. S3–S34 in the supplementary material. The appearance of the plots in Fig. 7, Fig. 8 are similar to those in Fig. 6, which simply shows that there is a close relationship between getting the fields right and getting the corrections right.

### Human data

Similarly to the simulated data, the human data also exhibited clear residual distortions after correcting for eddy currents, subject movement and a static susceptibility field. This manifested itself as changes in distortions between volumes, and can be seen as a movie in S2 in the supplementary material. It can also be seen how these apparent shape changes largely disappear when correcting also for susceptibility-by-movement effects.

The following are the supplementary data related to this article:

SimulatedMovie 1 SimulatedMovie

HumanMovie

#### Test-retest reproducibility of the estimated derivative fields

**Fig. 9:**
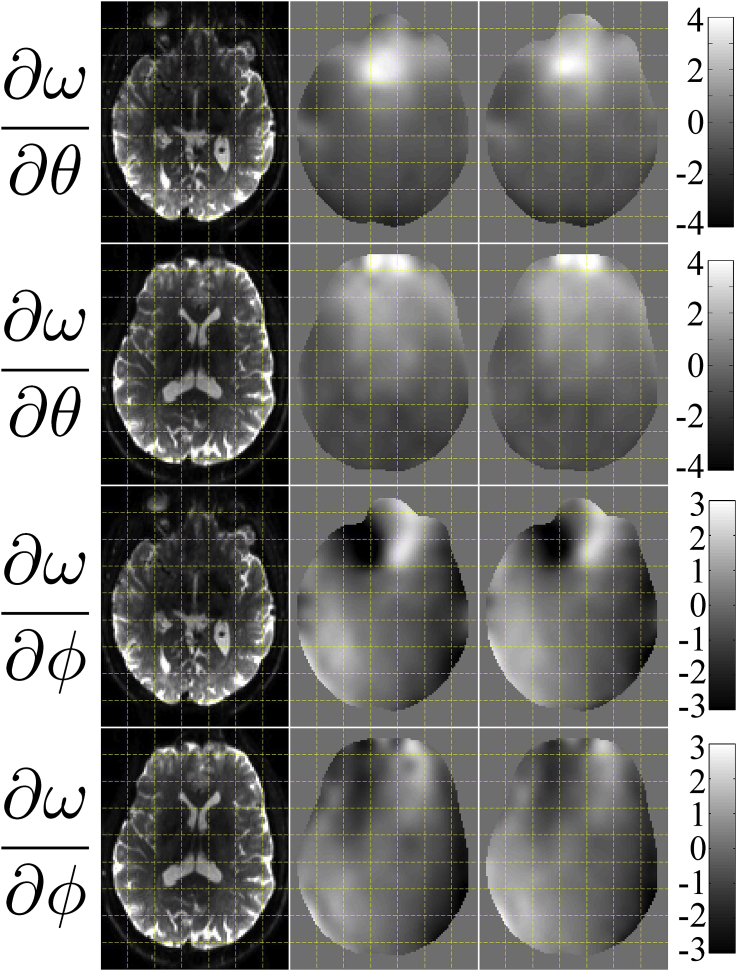
This figure shows the test-retest agreement of derivative fields estimated from two different data sets in the same subject. The first column is shown for anatomical reference and the two rightmost columns show the estimated derivative fields. The top two rows show the rate-of-change of the field with respect to pitch, and the bottom two rows with respect to roll. The units of the fields are Hz/degree rotation.

The outcome of estimating the derivative fields from two different runs is shown in Fig. 9. It can be seen that the reproducibility is good and the correlation between the two ∂ω/∂θ fields is 0.945 and for the ∂ω/∂ϕ fields it is 0.880. It can also be seen that there is a general agreement with the appearance of the derivative fields estimated from the simulated data (see Fig. 5). When considering Fig. 9 it should be remembered that the fields are estimated from completely distinct data sets with distinct subject movements and with a slightly different starting position of the subject.

#### Ability to estimate derivative fields from diffusion data

**Fig. 10:**
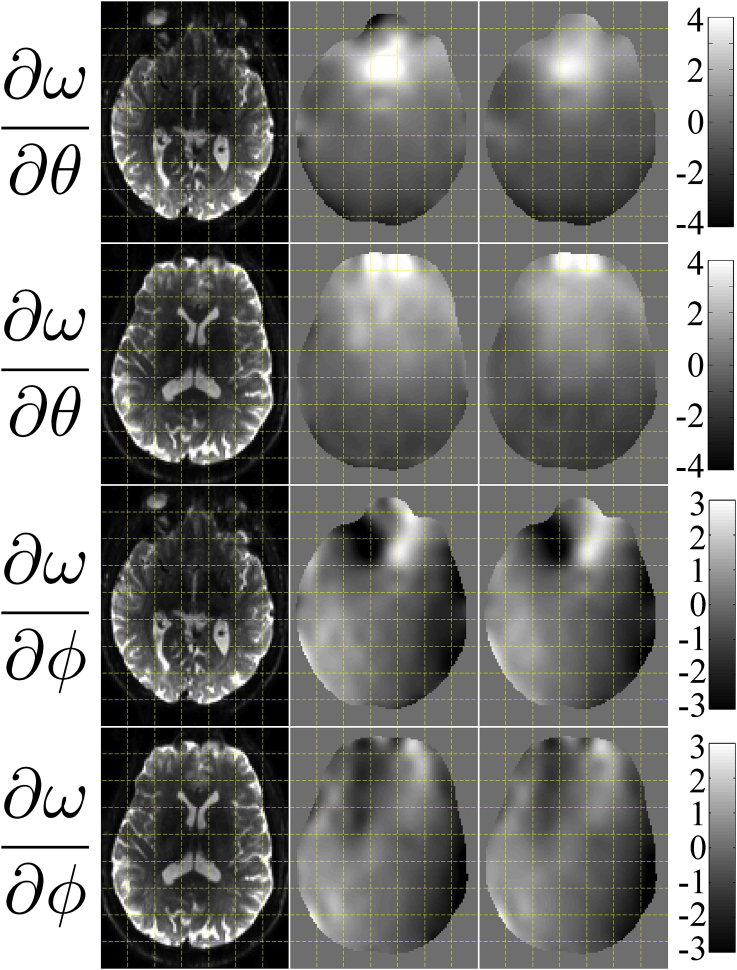
This figure shows the agreement between derivative fields estimated from b=0 volumes only (middle column) and from a diffusion data set (right column). The first column contains a corresponding b=0 volume after correction for susceptibility distortions, and is shown for anatomical reference. The top two rows show the rate-of-change of the field with respect to pitch, and the bottom two rows with respect to roll. The units of the fields are Hz/degree rotation.

The fields estimated from a diffusion data set (60 DWI + 5 b=0) compared to a field estimated from only b=0 volumes (61 vol) are shown in Fig. 10. The agreement is very good with a correlation of 0.966 for the ∂ω/∂θ fields and 0.975 for the ∂ω/∂ϕ fields.

#### Ability to correct the data for susceptibility-by-movement effects

**Fig. 11:**
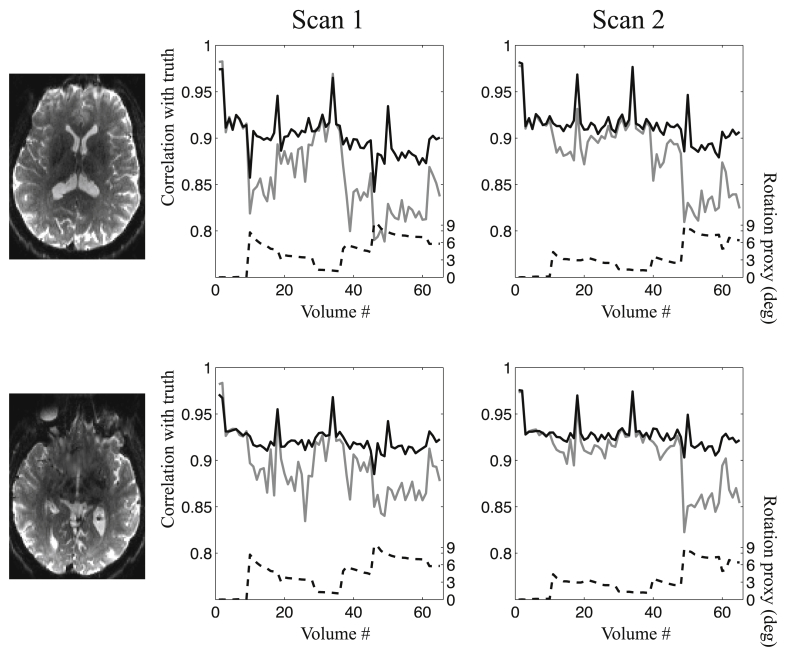
This figure shows the volume-wise correlation with “truth” when performing correction with (solid black line) and without (solid grey line) susceptibility-by-movement correction. The images to the left show the slices where the comparisons were made for the two rows. The leftmost plot pertains to the first scan and the rightmost to the second scan.

Fig. 11 shows a similar pattern to the simulated data with respect to our ability to correct the data. The greater our “rotation proxy” the greater the difference between the correction with and without susceptibility-by-movement correction. The overall correlations after correction are lower than for the simulated data. This would be expected since the rotation of the diffusion gradients in a subject framework leads to increasing differences in contrast with increasing subject rotation. We also find that the measured fields tend to be “sharper” (*i.e.* contain higher spatial frequencies) than the simulated ones, making their correction a little more difficult.

## Discussion

In this paper we have demonstrated our ability to track the movement-induced changes in the susceptibility-induced off-resonance field. It is very important to be clear that this is done without any need to actually measure any field, either by traditional field measurement or by reversed gradients. The method achieves this purely from the deviation of the observed images from the expected/predicted images. This is encouraging since it will enable workers to correct their data for this artefact without the need to alter protocols, acquire additional data or invest in costly hardware. It also means that the method can be applied to “historical” data that suffers from high levels of subject movement.

It should be noted that if a single, static, field is measured (for example by a volume pair with reversed gradients at the start of the scan) the expansion in equation (2) is performed with that field as the constant. In that case the corrected data will be internally consistent (corrected for susceptibility-by-movement effects) and anatomically faithful (corrected for susceptibility). If no susceptibility field is provided the constant term in equation (2) will be zero. The data will still be corrected for susceptibility-by-movement effects, and hence be internally consistent, but will not be anatomically faithful.

The results from our simulations and from our human experimental data are in good agreement, and indicate that the errors due to susceptibility-by-movement effects are determined by the amount of pitch and/or roll relative to the orientation of the subject when the field was measured. They also indicate that the method in the present paper can to a large degree eliminate this error. The rate-of-change maps are also in good agreement with those reported by Sulikowska (2016), both in magnitude and general appearance. Note that Sulikowska (2016) appears to have chosen an opposite convention with regards to the sign of the rotation (our fields are predicated on positive Δθ and Δϕ corresponding to nodding forward and tilting one's head to the right respectively), and also differ in terminology such that what we denote “roll” is denoted “yaw” in Sulikowska (2016).

### When do we need this?

The effects of the susceptibility field changes are relatively small in most of the brain and depend strongly on how much the subject moves. It is therefore of greatest importance for subjects who are uncooperative, such as babies, children or patients that find it hard to remain still. In this respect it is similar to corrections for intra-volume movement (Andersson et al., 2017), though the way its effects manifest themselves is very different. Intra-volume movement introduces variance in the data that is, as a first approximation, proportional to the temporal derivative of the movement. The changing susceptibility, in contrast, causes variance that is proportional to the movement, specifically to rotation around any axes non-parallel with the magnetic flux.

Our assumption is therefore that the present method will be important, together with the intra-volume motion correction (Andersson et al., 2017), for all categories of subjects that move a lot.

In addition we predict that it will be useful for very long examinations (such as for example the diffusion acquisitions in the HCP project, Sotiropoulos et al. (2013)), where even slow changes in position can add up to sizeable movements over the course of an hour or more. Since the susceptibility-by-movement effects are proportional to relative orientation it doesn't matter how slow the movement is and it can potentially have a significant impact on such studies.

### Higher *b*-values

In the present paper we have tested the method on simulated data with *b*-values up to 1000 and on human data with *b*-values up to 1500. It has previously been demonstrated (Andersson and Sotiropoulos, 2016) that eddy can correct for eddy current-induced distortions and subject movement for high *b*-value data. That ability hinges on its internal predictive model for the diffusion weighted images. We therefore expect this method to similarly work well on data up to at least the *b*-values of 5000 that was used in Andersson and Sotiropoulos (2016).

### Possible extensions

Another source of changing field is the breathing of the subject. Even though the lungs are a relatively long distance away from the object we image (the head) its effects on the field are experienced as a very smoothly varying off-resonance field at the location of the head. Such a smoothly varying field is in principle easy to compensate for with shimming, but the problem is that it varies during the respiratory cycle. Hence, it is another source of a field varying over time.

The framework we have suggested in the present paper can be extended to take this source into consideration. One would then replace (or add to) Δθ and Δϕ with a low-order basis expansion of the point in the respiratory cycle (for example measured using a bellows belt). This could be for example *p*, cos(πp) and cos(2πp) where *p* is a number between 0 and 1 defining the point in the respiratory cycle. It would also need to be changed so as to allow for different values of *p*, cos(πp) and cos(2πp) for different slices/multi-band-groups in Fig. 3 and equation (A8).

Our method could in principle also be combined with prospective motion correction (Maclaren et al., 2013) where subject movement is estimated by some external device (Maclaren et al., 2012) or by a 3D navigator (Alhamud et al., 2012) and used to inform the prescription of subsequent acquisitions. In that case the external recordings of pitch and roll would be used in lieu of the retrospectively estimated ones in equations (2), (8), (A8). It would also mean that the algorithm as described in 1 would be simplified in that no movement parameters would need to be estimated.

### Is it a problem that the derivative fields are poorly estimated when subjects move little?

No, it is not. It is the variance that is introduced by the susceptibility-by-movement that drives the estimation of the derivative fields. In the absence of susceptibility-by-movement-induced variance there is nothing to drive the estimation and it will be dominated by the regularisation of the fields, resulting in smooth estimated fields with values close to zero. When used for correction of the data (equation (2)) these “close to zero” fields will be multiplied by very small values for Δθ and Δϕ, and effectively not contribute at all. Which is the desired outcome when there is no susceptibility-by-movement-induced variance.

### Alternative methods

As far as we know the only viable alternatives to the method suggested in the present paper are the dynamic shimming methods suggested by Ward et al. (2002) and by Alhamud et al. (2016). The former of those methods was implemented for gradient echo EPI and has as far as we know not been applied to diffusion data. Furthermore, those methods are only capable of correcting for field changes that are constant or linear in space. As can be seen from Fig. 5, Fig. 9, Fig. 10 that is not sufficient to fully capture the actual field changes due to subject movement. Our tests (data not shown) indicate that a linear combination of linear fields in the principal directions can capture roughly half of the observed field changes. We don't know if either of those methods are freely available or implemented by any vendor.

The method suggested by Boegle et al. (2010) hinges on having a map of susceptibilities in exact register with the data one wishes to correct. Neither the acquisition or registration of such a map is trivial.

Methods based on tracking the phase of each voxel in the time-series (Ooi et al., 2013, Hutton et al., 2013, Dymerska et al., 2016) can not be used with spin-echo EPI data.

### Relation to earlier work

The ideas and principles behind the suggested method are similar to those described in Andersson et al. (2001), but there are many important differences.

• The inclusion of a predictive model for diffusion weighted images has allowed us to extend the method to diffusion data.
• The co-existence of volume-specific eddy current-induced fields with a changing susceptibility-induced field presents additional challenges that have been solved.
• It is implemented within a framework that models and corrects for intra-volume movement.
• The forward model has been complemented with an additional term (described as “Signal change caused by change in stretch/compression of signal” in Fig. 2) compared to the model that was used in Andersson et al. (2001).
• The use of a spline basis set (as opposed to the DCT set used in Andersson et al. (2001)) and the re-ordering of summations described in appendix C have facilitated estimating the derivative fields at higher spatial resolution with realistic computational and memory demands.

### Where to next?

In a series of papers we have described the development of a comprehensive tool to correct diffusion data for some of the most important sources of artefacts. The current version (as described in the present paper) corrects for eddy current-induced distortions, susceptibility-distortions (including changes with subject orientation) and subject movement (inter- and intra-volume bulk motion as well as motion induced signal drop-out). Additional sources that we hope will be addressed in future work include

**Spin history:** The signal in a given voxel depends on the time that has passed since it was previously excited, with shorter time leading to less signal. In the absence of movement this time is identical for all voxels in all volumes. When there is movement, that time can vary considerably and cause unwanted variance over volumes. With knowledge of the history of excitation for each voxel (which requires an intra-volume movement model) it should be possible to correct.

**Receive bias field:** There is, especially for coil arrays, an inhomogeneous intensity bias field caused by differential sensitivity to the RF-signal from different parts of the FOV. This is, to a first approximation, fixed in the scanner framework. When the subject moves within that, stationary, field it will result in unwanted variance across the volumes.

**Long time-constant eddy currents:** We have seen some evidence that the first one or two slices/MB-groups for a given volume (diffusion gradient) are affected by different eddy currents compared to the remaining slices/MB-groups. This violates the assumption within eddy of a single, constant, eddy current-induced field per volume.

## Conclusion

We have augmented our framework for simultaneous correction of susceptibility- and eddy current-induced distortions and subject movement effects with a model that estimates how the susceptibility-induced field changes with subject orientation.

## APPENDICES

### A Formal derivation of equations for the estimation

Equation (2) shows how to calculate the field for any orientation $r_{i}$ of the object. The unknowns in equation (2) are the derivative fields, *i.e.* $\partial \omega /\partial \theta {|}_{r_{0}}$, $\partial \omega /\partial \phi {|}_{r_{0}}$ etc. This section describes how to estimate these derivative fields.

We want to find the fields (∂ω/∂θ and ∂ω/∂ϕ) such that we minimise the mean squared difference between the GP predictions ${\overset{\text{˘}}{s}}_{i}$ and the corrected images ${\hat{s}}_{i}$. The summation is over all voxels in all volumes. If one assumes that the differences between the predicted and the corrected images are approximately normally distributed (*i.e.* that ε∼N(0,σ)) the least squares estimate would also be the maximum likelihood estimate of the fields. Hence we want to find the fields ∂ω that fulfills

(A1) $$\hat{\partial \omega }=\underset{\partial \omega }{\text{arg min}}C\left(\partial \omega \right)$$

where

(A2) $$C\left(\partial \omega \right)=\frac{1}{nN}\left[\begin{array}{c} {\overset{\text{˘}}{s}}_{1}-f_{1}\left(\partial \omega \right)⊙J_{1}\left(\partial \omega \right)\\ {\overset{\text{˘}}{s}}_{2}-f_{2}\left(\partial \omega \right)⊙J_{2}\left(\partial \omega \right)\\ \vdots \\ {\overset{\text{˘}}{s}}_{N}-f_{N}\left(\partial \omega \right)⊙J_{N}\left(\partial \omega \right) \end{array}\right]\left[\begin{array}{c} {\overset{\text{˘}}{s}}_{1}-f_{1}\left(\partial \omega \right)⊙J_{1}\left(\partial \omega \right)\\ {\overset{\text{˘}}{s}}_{2}-f_{2}\left(\partial \omega \right)⊙J_{2}\left(\partial \omega \right)\\ \vdots \\ {\overset{\text{˘}}{s}}_{N}-f_{N}\left(\partial \omega \right)⊙J_{N}\left(\partial \omega \right) \end{array}\right]$$

*i.e.* the mean squared difference between predictions and observations, where ∂ω denotes all the derivate fields in the model, *n* denotes the number of voxels in a volume, *N* denotes the number of volumes. $f_{i}\left(\partial \omega \right)$ denotes a column vector containing all *n* voxels of $f_{i}\left(\partial \omega ,\omega_{0},\beta_{i},r_{i},a_{i}\right)$, $J_{i}\left(\partial \omega \right)$ denotes all *n* voxels of $J\left(\partial \omega ,\omega_{0},\beta_{i},r_{i},a_{i}\right)$ and ⊙ denotes Hadamard product.

We have chosen to represent the fields as linear combinations of Cubic B-spline basis functions (Rueckert et al. (1999)), so for example ∂ω/∂θ is given by

(A3) $$\frac{\partial \omega }{\partial \theta }=Bb_{\theta }$$

where B is an n×M matrix of 3D-splines and where *M* is the number of splines used to represent a field. Hence, we can rewrite equations (A1), (A2) as

(A4) $$\hat{b}=\underset{b}{\text{arg min}}C\left(b\right)$$

where

(A5) $$C\left(b\right)=\frac{1}{nN}{\left[\begin{array}{c} {\overset{\text{˘}}{s}}_{1}-f_{1}\left(b\right)⊙J_{1}\left(b\right)\\ {\overset{\text{˘}}{s}}_{2}-f_{2}\left(b\right)⊙J_{2}\left(b\right)\\ \vdots \\ {\overset{\text{˘}}{s}}_{N}-f_{N}\left(b\right)⊙J_{N}\left(b\right) \end{array}\right]}^{\text{T}}\left[\begin{array}{c} {\overset{\text{˘}}{s}}_{1}-f_{1}\left(b\right)⊙J_{1}\left(b\right)\\ {\overset{\text{˘}}{s}}_{2}-f_{2}\left(b\right)⊙J_{2}\left(b\right)\\ \vdots \\ {\overset{\text{˘}}{s}}_{N}-f_{N}\left(b\right)⊙J_{N}\left(b\right) \end{array}\right]$$

where $b={\left[b_{\theta }^{\text{T}}\;b_{\phi }^{\text{T}}\right]}^{\text{T}}$.

The number of parameters (*i.e.* the size of b) can potentially be very large, so in order to estimate it we need an efficient optimisation method. We have opted for the Gauss-Newton method, which means b can be estimated from a sequence of iterations of the form

(A6) $$b^{\left(k+1\right)}=b^{\left(k\right)}-{\left(H{|}_{b^{\left(k\right)}}\right)}^{-1}\nabla {|}_{b^{\left(k\right)}}$$

where $\nabla {|}_{b^{\left(k\right)}}$ is the gradient of C calculated at $b^{\left(k\right)}$ and where $H{|}_{b^{\left(k\right)}}$ is the Gauss-Newton approximation to the Hessian calculated at the same point.

The gradient ∇ can be calculated as

(A7) $$\nabla =-\frac{2}{nN}\underset{\begin{array}{c} X \end{array}}{\underset{︸}{\left[\begin{array}{c} \frac{d}{db}\left[f_{1}\left(b\right)\right]⊙\left(1^{\text{T}}⊗J_{1}\left(b\right)\right)+\left(1^{\text{T}}⊗f_{1}\left(b\right)\right)⊙\frac{d}{db}\left[J_{1}\left(b\right)\right]\\ \frac{d}{db}\left[f_{2}\left(b\right)\right]⊙\left(1^{\text{T}}⊗J_{2}\left(b\right)\right)+\left(1^{\text{T}}⊗f_{2}\left(b\right)\right)⊙\frac{d}{db}\left[J_{2}\left(b\right)\right]\\ \vdots \\ \frac{d}{db}\left[f_{N}\left(b\right)\right]⊙\left(1^{\text{T}}⊗J_{N}\left(b\right)\right)+\left(1^{\text{T}}⊗f_{N}\left(b\right)\right)⊙\frac{d}{db}\left[J_{N}\left(b\right)\right] \end{array}\right]}}\left[\begin{array}{c} {\overset{\text{˘}}{s}}_{1}-f_{1}\left(b\right)⊙J_{1}\left(b\right)\\ {\overset{\text{˘}}{s}}_{2}-f_{2}\left(b\right)⊙J_{2}\left(b\right)\\ \vdots \\ {\overset{\text{˘}}{s}}_{N}-f_{N}\left(b\right)⊙J_{N}\left(b\right) \end{array}\right]$$

where ⊗ denotes the Kronecker product. The components of the first term in X are as follows: $1^{\text{T}}⊗J_{i}\left(b\right)$ is simply 2M (2 because we are modeling 2 fields) copies of the Jacobian volume $J_{i}$ concatenated horizontally.

$\frac{d}{db}\left[f_{i}\left(b\right)\right]$ is defined as

(A8) $$\frac{d}{db}\left[f_{i}\left(b\right)\right]=\left[\begin{array}{cc} \text{Δ}\theta_{i}\frac{d}{db_{\theta }}\left[f_{i}\left(b\right)\right] & \text{Δ}\phi_{i}\frac{d}{db_{\phi }}\left[f_{i}\left(b\right)\right] \end{array}\right]$$

where

(A9) $$\frac{d}{db_{\theta }}\left[f_{i}\left(b\right)\right]=\left[\begin{array}{cccc} \frac{\partial f_{i}^{\left(1\right)}}{\partial b_{\theta }^{\left(1\right)}} & \frac{\partial f_{i}^{\left(1\right)}}{\partial b_{\theta }^{\left(2\right)}} & \cdots & \frac{\partial f_{i}^{\left(1\right)}}{\partial b_{\theta }^{\left(M\right)}}\\ \frac{\partial f_{i}^{\left(2\right)}}{\partial b_{\theta }^{\left(1\right)}} & \frac{\partial f_{i}^{\left(2\right)}}{\partial b_{\theta }^{\left(2\right)}} & \cdots & \frac{\partial f_{i}^{\left(2\right)}}{\partial b_{\theta }^{\left(M\right)}}\\ \vdots & \vdots & \ddots & \vdots \\ \frac{\partial f_{i}^{\left(n\right)}}{\partial b_{\theta }^{\left(1\right)}} & \frac{\partial f_{i}^{\left(n\right)}}{\partial b_{\theta }^{\left(2\right)}} & \cdots & \frac{\partial f_{i}^{\left(n\right)}}{\partial b_{\theta }^{\left(M\right)}} \end{array}\right]$$

and where $\partial f_{i}^{\left(j\right)}/\partial b_{\theta }^{\left(k\right)}$ is the partial derivative of the *j*th voxel of the *i*th volume w.r.t. the *k*th element of $b_{\theta }$. $\frac{d}{db_{\phi }}\left[f_{i}\left(b\right)\right]$ is defined correspondingly. Finally $\frac{\partial f_{i}^{\left(j\right)}}{\partial b^{\left(k\right)}}$ is given by

(A10) $$\frac{\partial f_{i}^{\left(j\right)}}{\partial b^{\left(k\right)}}=t_{i}\left(p_{i}^{x}\frac{\partial f_{i}^{\left(j\right)}}{\partial x}+p_{i}^{y}\frac{\partial f_{i}^{\left(j\right)}}{\partial y}\right)B_{jk}$$

where $t_{i}$ is the total readout time for a slice/group of the *i*th volume, $p_{i}^{x}$ and $p_{i}^{y}$ are the *x*- and *y*-components of the phase-encode direction of the *i*th volume and $f_{i}^{\left(j\right)}/\partial x$ and $f_{i}^{\left(j\right)}/\partial y$ are the *x*- and *y*-components of the image gradient of $f_{i}$ at voxel *j*.

Among the components of the second term, $\left(1^{\text{T}}⊗f_{i}\left(b\right)\right)$ is 2M copies of the (corrected) image $f_{i}$. $\frac{d}{db_{\theta }}\left[J_{i}\left(b\right)\right]$ is given by

(A11) $$\frac{d}{db_{\theta }}\left[J_{i}\left(b\right)\right]=\left[\begin{array}{cccc} \frac{\partial J_{i}^{\left(1\right)}}{\partial b_{\theta }^{\left(1\right)}} & \frac{\partial J_{i}^{\left(1\right)}}{\partial b_{\theta }^{\left(2\right)}} & \cdots & \frac{\partial J_{i}^{\left(1\right)}}{\partial b_{\theta }^{\left(M\right)}}\\ \frac{\partial J_{i}^{\left(2\right)}}{\partial b_{\theta }^{\left(1\right)}} & \frac{\partial J_{i}^{\left(2\right)}}{\partial b_{\theta }^{\left(2\right)}} & \cdots & \frac{\partial J_{i}^{\left(2\right)}}{\partial b_{\theta }^{\left(M\right)}}\\ \vdots & \vdots & \ddots & \vdots \\ \frac{\partial J_{i}^{\left(n\right)}}{\partial b_{\theta }^{\left(1\right)}} & \frac{\partial J_{i}^{\left(n\right)}}{\partial b_{\theta }^{\left(2\right)}} & \cdots & \frac{\partial J_{i}^{\left(n\right)}}{\partial b_{\theta }^{\left(M\right)}} \end{array}\right]$$

where

(A12) $$\frac{\partial J_{i}^{\left(j\right)}}{\partial b^{\left(k\right)}}=t_{i}\left(p_{i}^{x}B_{jk}^{x}+p_{i}^{y}B_{jk}^{y}\right)$$

and where $t_{i}$, $p_{i}^{x}$ and $p_{i}^{y}$ are defined as in equation (A10) and where $B^{x}$ and $B^{y}$ are matrices of 3D-splines differentiated in the *x*- and *y*-directions respectively.

Using equations (A8), (A9), (A10), (A11), (A12) one can calculate X, and inserting that into equation (A7) gives the gradient ∇. The Gauss-Newton approximation of the Hessian is simply $H=X^{\text{T}}X$, hence we have all we need for the iterative process indicated by equation (A6) to estimate the derivative fields.

#### A.1 Regularisation of the field

To ensure that the estimated derivative fields are well behaved we include a regularisation term such that the cost-function in equation (A5) becomes

(A13) $$\underset{b}{\text{arg min}}C\left(b\right)=\frac{1}{nN}{\left[\begin{array}{c} {\overset{\text{˘}}{s}}_{1}-f_{1}\left(b\right)⊙J_{1}\left(b\right)\\ {\overset{\text{˘}}{s}}_{2}-f_{2}\left(b\right)⊙J_{2}\left(b\right)\\ \vdots \\ {\overset{\text{˘}}{s}}_{N}-f_{N}\left(b\right)⊙J_{N}\left(b\right) \end{array}\right]}^{\text{T}}\left[\begin{array}{c} {\overset{\text{˘}}{s}}_{1}-f_{1}\left(b\right)⊙J_{1}\left(b\right)\\ {\overset{\text{˘}}{s}}_{2}-f_{2}\left(b\right)⊙J_{2}\left(b\right)\\ \vdots \\ {\overset{\text{˘}}{s}}_{N}-f_{N}\left(b\right)⊙J_{N}\left(b\right) \end{array}\right]+\frac{1}{m}\sum_{i=1}^{m}\lambda b_{i}^{\text{T}}Sb_{i}$$

where *m* is the number of fields (m=2 for the case of a first order expansion with respect to Δθ and Δϕ), $b_{i}$ is the part of the vector b that pertains to the *i*th field and where S is a matrix that implements the bending energy of a field given by $b_{i}$.

### B Extension to a second order model

When deriving the method in sections 3.1–3.4 of the main text and appendix A we have mostly (with the exception of equation (2)) used a first order Taylor expansion of the susceptibility-induced field as our example. This was done to provide an example that was not overly complicated, and that would also allow us to fit the equations within the margins of the pages. In this appendix we will show how to extend the estimation to a second order model. A second order model is also what has been implemented in our software eddy, with the option to limit it to a first order model (that is also the default).

Furthermore, in the main text we only considered changes of the field caused by rotations (*θ* and *ϕ*) around the axes orthogonal to the magnetic flux (*x* and *y*). This is in accordance with the theory, but mathematically there is nothing to stop one from modeling the changes w.r.t. any of the rigid-body movement parameters, thus testing that theory. This is how it has been implemented in eddy, and the user has a choice as to which of the movement parameters to include in the model (though the default is *θ* and *ϕ*).

If we denote the subset of movement parameters we want to include in the model by r and the number of parameters as *n* we can write equation (2) as

(B1) $$\omega \left(x:r\right)=\omega \left(x:r^{\left(0\right)}\right)+\text{Δ}r_{1}\frac{\partial \omega }{\partial r_{1}}+\cdots +\text{Δ}r_{n}\frac{\partial \omega }{\partial r_{n}}+\frac{\text{Δ}r_{1}^{2}}{2}\frac{\partial^{2}\omega }{\partial r_{1}^{2}}+\cdots +\frac{\text{Δ}r_{n-1}\text{Δ}r_{n}}{2}\frac{\partial^{2}\omega }{\partial r_{n-1}\partial r_{n}}+R_{2}$$

where we have denoted the orientation of the subject at which the field was measured as $r^{\left(0\right)}$ and where it is implicit that all the derivative fields are defined at that same point.

This model contains n+n(n+1)/2 fields that need to be estimated, so for example in the case with only *θ* and *ϕ* the number of fields would be five and if modeling all six movement parameters it would be (a forbidding) 27. The iterative procedure now becomes

$$b^{0}=0$$

$$y^{\left(j\right)}=\left[\begin{array}{c} {\overset{\text{˘}}{s}}_{0}^{\left(j\right)}-f_{0}^{\left(j\right)}\\ {\overset{\text{˘}}{s}}_{1}^{\left(j\right)}-f_{1}^{\left(j\right)}\\ \vdots \\ {\overset{\text{˘}}{s}}_{N-1}^{\left(j\right)}-f_{N-1}^{\left(j\right)} \end{array}\right]$$

(B2) $$X^{\left(j\right)}=\left[\begin{array}{cccccc} \text{Δ}r_{\left(1,1\right)}D_{1}^{\left(j\right)} & \cdots & \text{Δ}r_{\left(n,1\right)}D_{1}^{\left(j\right)} & \frac{\text{Δ}r_{\left(1,1\right)}^{2}}{2}D_{1}^{\left(j\right)} & \cdots & \frac{\text{Δ}r_{\left(n-1,1\right)}\text{Δ}r_{\left(n,1\right)}}{2}D_{1}^{\left(j\right)}\\ \text{Δ}r_{\left(1,2\right)}D_{2}^{\left(j\right)} & \cdots & \text{Δ}r_{\left(n,2\right)}D_{2}^{\left(j\right)} & \frac{\text{Δ}r_{\left(1,2\right)}^{2}}{2}D_{2}^{\left(j\right)} & \cdots & \frac{\text{Δ}r_{\left(n-1,2\right)}\text{Δ}r_{\left(n,2\right)}}{2}D_{2}^{\left(j\right)}\\ \vdots & \ddots & \vdots & \vdots & \ddots & \vdots \\ \text{Δ}r_{\left(1,N\right)}D_{N}^{\left(j\right)} & \cdots & \text{Δ}r_{\left(n,N\right)}D_{N}^{\left(j\right)} & \frac{\text{Δ}r_{\left(1,N\right)}^{2}}{2}D_{N}^{\left(j\right)} & \cdots & \frac{\text{Δ}r_{\left(n-1,N\right)}\text{Δ}r_{\left(n,N\right)}}{2}D_{N}^{\left(j\right)} \end{array}\right]$$

$$b^{\left(j+1\right)}=b^{\left(j\right)}+{\left({\left(X^{\left(j\right)}\right)}^{\text{T}}X^{\left(j\right)}\right)}^{-1}{\left(X^{\left(j\right)}\right)}^{\text{T}}y^{\left(j\right)}$$

where $r_{\left(i,j\right)}$ denotes the *i*th movement parameter for the *j*th volume and where

(B3) $$D_{i}^{\left(j\right)}=\left(1^{\text{T}}⊗\left[t_{i}\left(p_{i}^{x}\frac{\partial f_{i}}{\partial x}{|}_{\omega_{i}^{\left(j\right)}}+p_{i}^{y}\frac{\partial f_{i}}{\partial y}{|}_{\omega_{i}^{\left(j\right)}}\right)⊙J\left(\omega_{i}^{\left(j\right)},\beta_{i},r_{i},a_{i}\right)\right]\right)⊙B$$

### C Computational complexity

There is one problem with equations (A8), (A9), (A10), (A11), (A12) as they stand. The computational cost of calculating $X^{\text{T}}X$ is huge. The size of X is $NN_{x}N_{y}N_{z}\times LM$ where *N* is the number of volumes, $N_{x}$, $N_{y}$ and $N_{z}$ define the dimensions of a single volume, *M* is the number of splines used to model a field and *L* depends on the order of the Taylor expansion in equation (2).

If we take an HCP data set (Sotiropoulos et al. (2013)) as an example $NN_{x}N_{y}N_{z}=1,546,739,712$. *L* would be 2 for the lowest (first) order expansion with respect to *θ* and *ϕ*. Finally, if we assume a warp resolution of 10 mm for the fields, M=8064. Hence, $X^{\text{T}}X$ has 130,064,256 unique elements, each of which takes 9,280,438,272 multiplications and 4,640,219,136 additions to calculate. Even for a GPU, a straightforward calculation of $X^{\text{T}}X$ is out of the question.

Luckily there are ways to increase the efficiency of the calculations. If we use the lowest order expansion as an example, $X^{\text{T}}X$ it is of the form

(C1) $$X^{\text{T}}X=\left[\begin{array}{cc} \sum_{i=1}^{N}X_{\theta ,i}^{\text{T}}X_{\theta ,i} & \sum_{i=1}^{N}X_{\theta ,i}^{\text{T}}X_{\phi ,i}\\ \sum_{i=1}^{N}X_{\phi ,i}^{\text{T}}X_{\theta ,i} & \sum_{i=1}^{N}X_{\phi ,i}^{\text{T}}X_{\phi ,i} \end{array}\right]$$

where for example $X_{\theta ,i}$ refers to the sub-matrix of X (equation (A7)) that pertains to volume *i* and the ∂ω/∂θ field. The $X^{\text{T}}X$ in equation (C1) consists of four sub-matrices where for example

(C2) $$\overset{N}{\sum_{i=1}}X_{\theta }^{\text{T}}X_{\theta }=\overset{N}{\sum_{i=1}}{\left(\text{Δ}\theta_{i}B⊙\partial f_{i}\right)}^{\text{T}}\left(\text{Δ}\theta_{i}B⊙\partial f_{i}\right)+\overset{N}{\sum_{i=1}}{\left(\text{Δ}\theta_{i}{B^{'}}_{i}⊙f_{i}\right)}^{\text{T}}\left(\text{Δ}\theta_{i}{B^{'}}_{i}⊙f_{i}\right)+\overset{N}{\sum_{i=1}}{\left(\text{Δ}\theta_{i}{B^{'}}_{i}⊙f_{i}\right)}^{\text{T}}\left(\text{Δ}\theta_{i}B⊙\partial f_{i}\right)+\overset{N}{\sum_{i=1}}{\left(\text{Δ}\theta_{i}B⊙\partial f_{i}\right)}^{\text{T}}\left(\text{Δ}\theta_{i}{B^{'}}_{i}⊙f_{i}\right)$$

where $f_{i}$ denotes $1^{\text{T}}⊗f_{i}$ (*i.e. M* copies of $f_{i}$ horizontally concatenated), where

(C3) $$\partial f_{i}=1^{\text{T}}⊗t_{i}\left(p_{i}^{x}\frac{\partial f_{i}}{\partial x}+p_{i}^{y}\frac{\partial f_{i}}{\partial y}\right)$$

and

(C4) $${B^{\prime }}_{i}=t_{i}\left(p_{i}^{x}B^{x}+p_{i}^{y}B^{y}\right)$$

When there is a limited number of different values of $p_{i}^{x}$ and $p_{i}^{y}$, this can easily be re-written to achieve almost a factor of *N* speed up. For the following example let us assume a data set where phase encoding is in the *y*-direction for all volumes so that $p_{i}^{x}=0$ for all volumes *i* and so that $p_{i}^{y}$ is always either 1 or -1. The matrix in equation C2 can then be rewritten as

(C5) $$\overset{N}{\sum_{i=1}}X_{\theta }^{\text{T}}X_{\theta }=B^{\text{T}}\left(B⊙\left(\overset{N}{\sum_{i=1}}\text{Δ}\theta_{i}^{2}\partial f_{i}⊙\partial fi\right)\right)+{\left(B^{y}\right)}^{\text{T}}\left(B^{y}⊙\left(\overset{N}{\sum_{i=1}}\text{Δ}\theta_{i}^{2}t_{i}^{2}f_{i}⊙f_{i}\right)\right)+{\left(B^{y}\right)}^{\text{T}}\left(B⊙\left(\overset{N}{\sum_{i=1}}\text{Δ}\theta_{i}^{2}t_{i}f_{i}⊙\partial f_{i}\right)\right)+B^{\text{T}}\left(B^{y}⊙\left(\overset{N}{\sum_{i=1}}\text{Δ}\theta_{i}^{2}t_{i}\partial f_{i}⊙f_{i}\right)\right)$$

There are additional re-orderings that can be, and are, used to speed up the calculations, but this is the most important one. Furthermore, the resulting matrix $X^{\text{T}}X$ is very sparse (the higher the warp resolution, the sparser $X^{\text{T}}X$ becomes) which can also be used to speed up its calculation.
