# Supplementary material for: Susceptibility-induced distortion that varies due to motion: Correction in diffusion MR without acquiring additional data
Source: Neuroimage. 2018 May 1;171:277–95. doi: 10.1016/j.neuroimage.2017.12.040 (PMC5883370; doi:10.1016/j.neuroimage.2017.12.040)
Supplement: Supplementary data [file mmc3.pdf]

so that $p_i^y$ is always either 1 or -1. The matrix in equation C2 can then be rewritten as

$$\sum_{i=1}^{N} \mathbf{X}_\theta^{\mathrm{T}} \mathbf{X}_\theta = \mathbf{B}^{\mathrm{T}} \left( \mathbf{B} \odot \left( \sum_{i=1}^{N} \Delta\theta_i^2 \partial f_i \odot \partial fi \right) \right) + (\mathbf{B}^y)^{\mathrm{T}} \left( \mathbf{B}^y \odot \left( \sum_{i=1}^{N} \Delta\theta_i^2 t_i^2 f_i \odot f_i \right) \right) + (\mathbf{B}^y)^{\mathrm{T}} \left( \mathbf{B} \odot \left( \sum_{i=1}^{N} \Delta\theta_i^2 t_i f_i \odot \partial f_i \right) \right) + \mathbf{B}^{\mathrm{T}} \left( \mathbf{B}^y \odot \left( \sum_{i=1}^{N} \Delta\theta_i^2 t_i \partial f_i \odot f_i \right) \right) \tag{C5}$$

There are additional re-orderings that can be, and are, used to speed up the calculations, but this is the most important one. Furthermore, the resulting matrix $\mathbf{X}^{\mathrm{T}}\mathbf{X}$ is very sparse (the higher the warp resolution, the sparser $\mathbf{X}^{\mathrm{T}}\mathbf{X}$ becomes) which can also be used to speed up its calculation.

# S Supplementary material

Listed below are tables and figures that were deemed superfluous to the main article.

## S.1 Movies

A very convincing way of demonstrating the susceptibility-by-movement effect, and the ability of the present method to correct for it, is by vieweing the data as a movie. Below we show movies with and without correction for susceptibility-by-movement for the simulated and for the human data.

### S.1.1 Simulations

Figure S1 is a still from the movie SimulatedMovie.gif.

### S.1.2 Human data

Figure S2 is a still from the movie HumanMovie.gif.

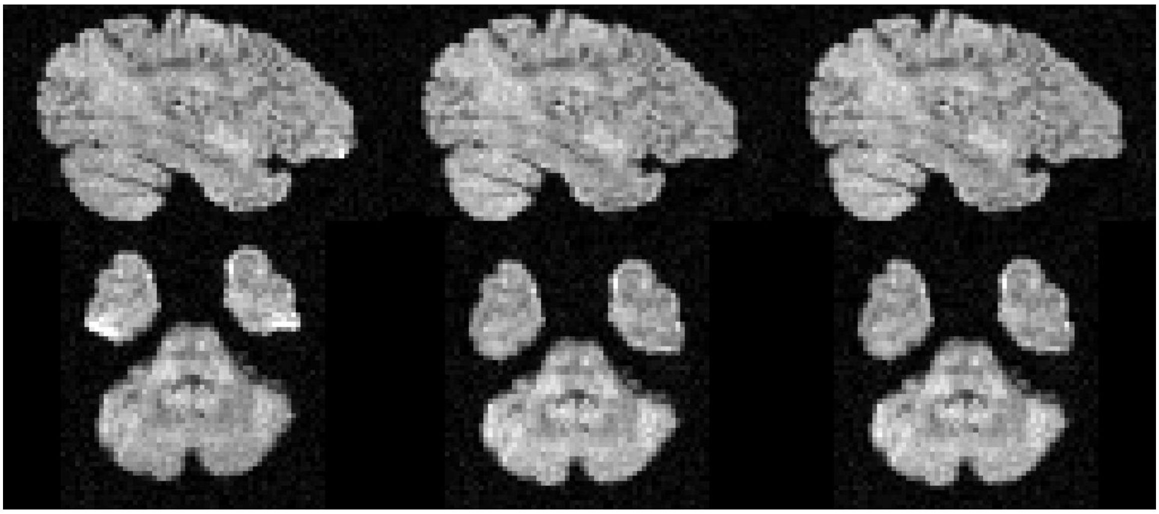

Figure S1: The movie shows data before correction (leftmost column) and after correction for susceptibility, eddy currents and subject movement (middle column). The rightmost column shows the data after including also the susceptibility-by-movement model in the correction.

## S.2 Correlations between true and estimated derivative maps for simulated data

Figure 5 shows a side-by-side comparison of the “true” and estimated and derivative fields for two specific sets of simulations. Below we have tabulated (tables S1 and S2) the correlation between “true” and estimated maps for all sets of simulations. To convey an intuition of what these correlations mean we mention that the correlations of the maps in columns 3 and 4 with that in column 2 of figure 5 were 0.864 and 0.864 respectively for $\partial\omega/\partial\theta$ and 0.878 and 0.879 respectively for $\partial\omega/\partial\phi$. The corresponding numbers for columns 6 and 7 with column 5 were 0.957 and 0.957 for $\partial\omega/\partial\theta$ and 0.954 and 0.954 for $\partial\omega/\partial\phi$.

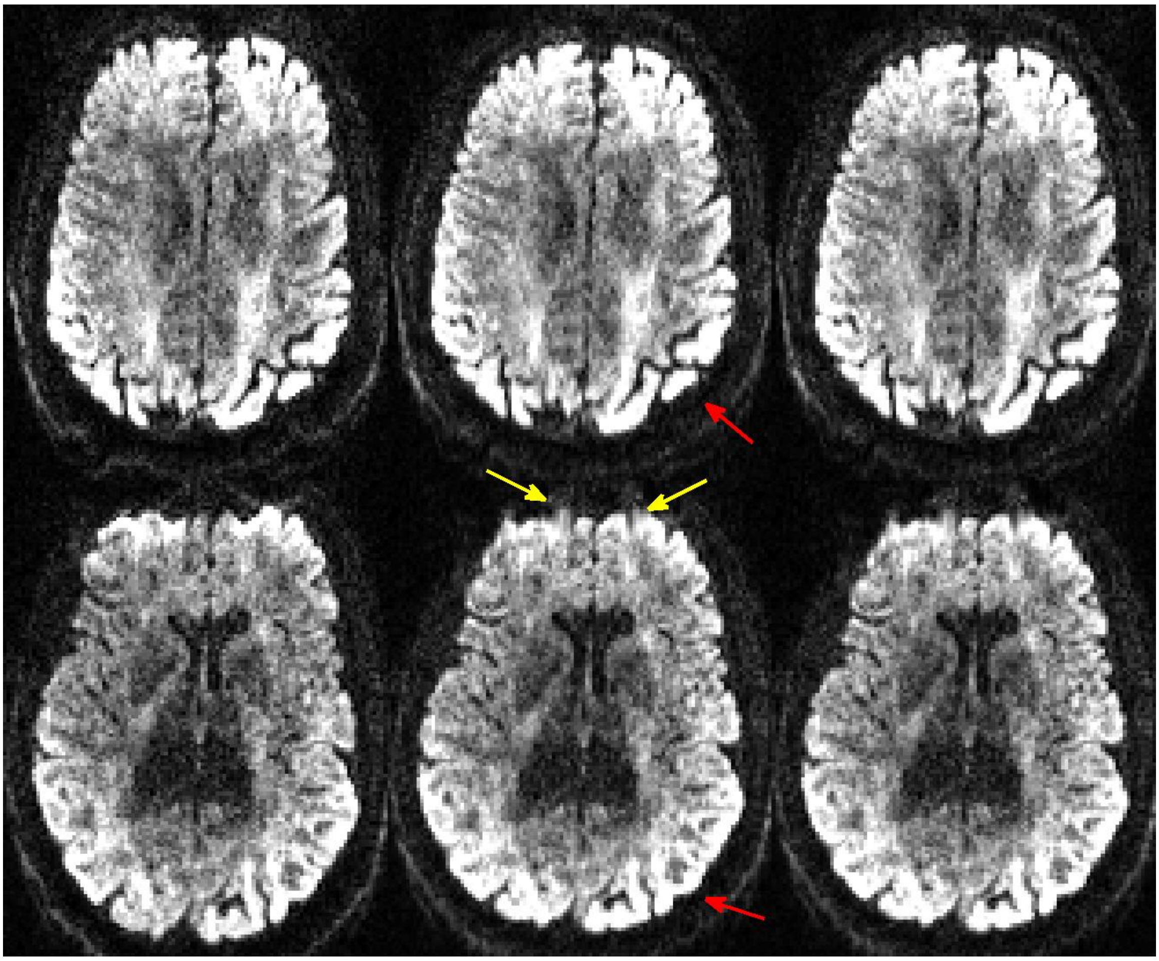

Figure S2: The movie shows data before correction (leftmost column) and after correction for susceptibility, eddy currents and subject movement (middle column). The rightmost column shows the data after including also the susceptibility-by-movement model in the correction. The yellow arrows points to an area with rapid spatial changes in the susceptibility-field. The red arrows point to areas with much smoother susceptibility-field, but which nevertheless changes when the subject moves.

| | | | | | | | | |
|---|---|---|---|---|---|---|---|---|
| Movement type: | Volumetric | | | | | | | |
| PE-direction: | A→P | | | | | | | |
| Derivative type: | $\partial\omega/\partial\theta$ | | | | | | | |
| Eddy currents: | Without | | | | With | | | |
| Movement magnitude: | Normal | | Large | | Normal | | Large | |
| SNR: | 20 | 40 | 20 | 40 | 20 | 40 | 20 | 40 |
| Correlation: | .859 | .864 | .955 | .957 | .860 | .864 | .955 | .957 |
| Derivative type: | $\partial\omega/\partial\phi$ | | | | | | | |
| Eddy currents: | Without | | | | With | | | |
| Movement magnitude: | Normal | | Large | | Normal | | Large | |
| SNR: | 20 | 40 | 20 | 40 | 20 | 40 | 20 | 40 |
| Correlation: | .873 | .878 | .952 | .954 | .874 | .879 | .953 | .954 |
| PE-direction: | P→A | | | | | | | |
| Derivative type: | $\partial\omega/\partial\theta$ | | | | | | | |
| Eddy currents: | Without | | | | With | | | |
| Movement magnitude: | Normal | | Large | | Normal | | Large | |
| SNR: | 20 | 40 | 20 | 40 | 20 | 40 | 20 | 40 |
| Correlation: | .848 | .860 | .953 | .956 | .854 | .861 | .952 | .955 |
| Derivative type: | $\partial\omega/\partial\phi$ | | | | | | | |
| Eddy currents: | Without | | | | With | | | |
| Movement magnitude: | Normal | | Large | | Normal | | Large | |
| SNR: | 20 | 40 | 20 | 40 | 20 | 40 | 20 | 40 |
| Correlation: | .868 | .878 | .950 | .951 | .870 | .878 | .950 | .951 |

Table S1: The table shows the correlation between "true" and estimated derivative fields for simulated data with "volumetric" movement. Each number is the average of ten unique noise realisations. The standard deviation was less than 0.001 for all cases, so we have chosen not to tabulate it.

| | | | | | | | | |
|---|---|---|---|---|---|---|---|---|
| Movement type: | Slice-wise | | | | | | | |
| PE-direction: | A→P | | | | | | | |
| Derivative type: | $\partial\omega/\partial\theta$ | | | | | | | |
| Eddy currents: | Without | | | | With | | | |
| Movement magnitude: | Normal | | Large | | Normal | | Large | |
| SNR: | 20 | 40 | 20 | 40 | 20 | 40 | 20 | 40 |
| Correlation: | .856 | .862 | .953 | .955 | .855 | .860 | .954 | .955 |
| Derivative type: | $\partial\omega/\partial\phi$ | | | | | | | |
| Eddy currents: | Without | | | | With | | | |
| Movement magnitude: | Normal | | Large | | Normal | | Large | |
| SNR: | 20 | 40 | 20 | 40 | 20 | 40 | 20 | 40 |
| Correlation: | .868 ± .001 | .875 | .950 | .952 | .868 | .875 | .950 | .952 |
| PE-direction: | P→A | | | | | | | |
| Derivative type: | $\partial\omega/\partial\theta$ | | | | | | | |
| Eddy currents: | Without | | | | With | | | |
| Movement magnitude: | Normal | | Large | | Normal | | Large | |
| SNR: | 20 | 40 | 20 | 40 | 20 | 40 | 20 | 40 |
| Correlation: | .849 | .860 | .951 | .955 | .844 ± .002 | .856 | .950 | .955 |
| Derivative type: | $\partial\omega/\partial\phi$ | | | | | | | |
| Eddy currents: | Without | | | | With | | | |
| Movement magnitude: | Normal | | Large | | Normal | | Large | |
| SNR: | 20 | 40 | 20 | 40 | 20 | 40 | 20 | 40 |
| Correlation: | .854 ± .002 | .868 ± .003 | .946 | .949 | .850 ± .004 | .867 ± .003 | .946 | .949 |

Table S2: The table shows the correlation between "true" and estimated derivative fields for simulated data with "volumetric" movement. Each number is the average of ten unique noise realisations. For the cases where the standard deviation was less than 0.001 we have chosen not to tabulate it.

## S.3 Comparing corrected and true images for simulated data

Figures 7 and 8 in the main paper show the correlation between true and corrected images for a quite basal slice, a $A \rightarrow P$ PE-direction and an SNR of 40. In this section we show similar plots for different levels (in the $z$-direction) in the brain, for both inter- and intra-volume movement and for PE in both $A \rightarrow P$ and $P \rightarrow A$ directions and for an SNR of 20 and 40.

For all figures in this section the solid black and solid grey lines represent the correlation between true and corrected images for the method in the present paper and the method assuming a constant susceptibility field respectively. The scale for both these curves is found on the left-hand of the graphs. The dashed black line shows a "proxy" for the rotation relevant to susceptibility field. It was calculated as $\sqrt{R_x^2 + R_y^2}$ where $R_x$ and $R_y$ denote rotation around the $x$- and $y$-axes respectively. The scale for that curve is found on the right hand side of the graphs. The graphs in the left column show the results for "normal" subject movement and the right column for "large" movement. The top row shows the situation when no eddy current distortions were simulated and no attempt was made to estimate eddy currents. The bottom row shows the situation when eddy currents were included in the simulations and eddy currents and susceptibility-by-movement were jointly estimated by `eddy`.

The general appearance of all these plots is similar, but with some potentially interesting messages in the differences between them.

- The gains from correcting for susceptibility-by-movement depend strongly on the parts of the brain measured, with regions close to the sinuses or the ear canals most affected.
- There does not seem to be any difference between PE in $A \rightarrow P$ or in $P \rightarrow A$ when it comes to our ability to correct for susceptibility-by-movement effects.
- The presence of eddy currents and intra-volume movement seems to have a small but

appreciable negative impact on our ability to correct for susceptibility-by-movement effects.

- When assessed over the whole brain the correction has a positive impact for all cases in our $2 \times 2 \times 2 \times 2 \times 2$ factorial.

#### S.3.0.1 Simulations with only inter-volume (volumetric) movement.

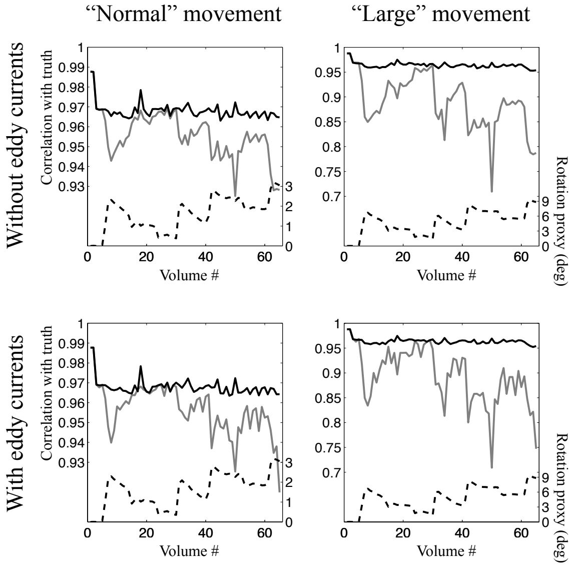


Figure S3: Results for slice 15 of simulations with volumetric movement, PE in the $A \to P$ direction and an SNR of 20

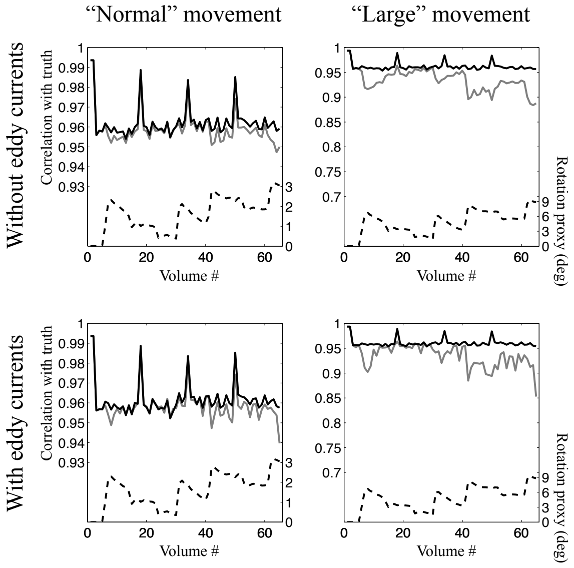


Figure S4: Results for slice 25 of simulations with volumetric movement, PE in the $A \to P$ direction and an SNR of 20

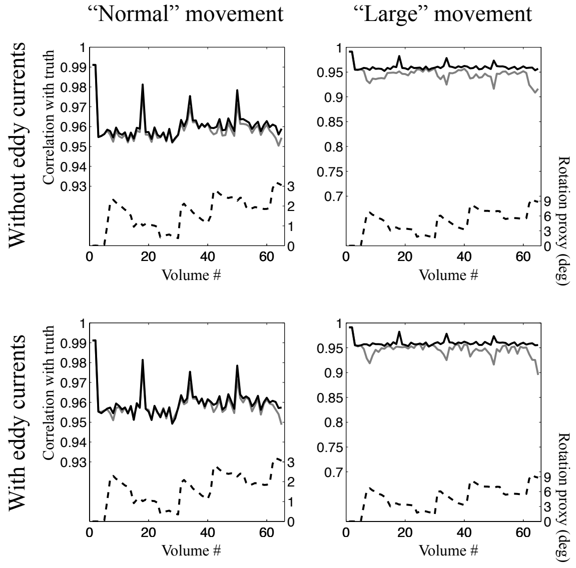


Figure S5: Results for slice 35 of simulations with volumetric movement, PE in the $A \rightarrow P$ direction and an SNR of 20

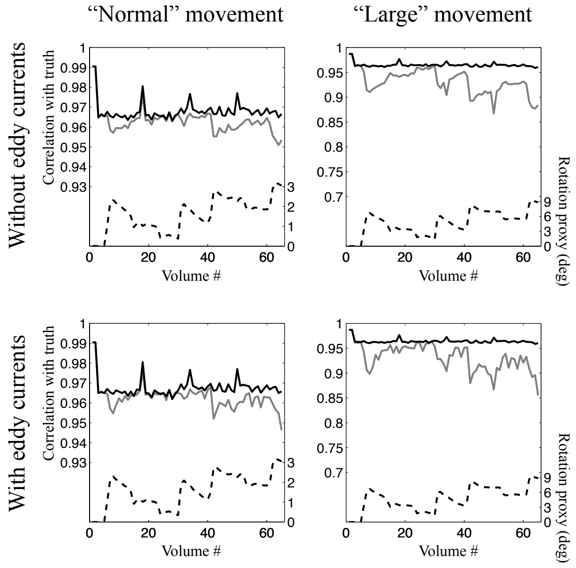


Figure S6: Results for the whole brain of simulations with volumetric movement, PE in the $A \rightarrow P$ direction and an SNR of 20

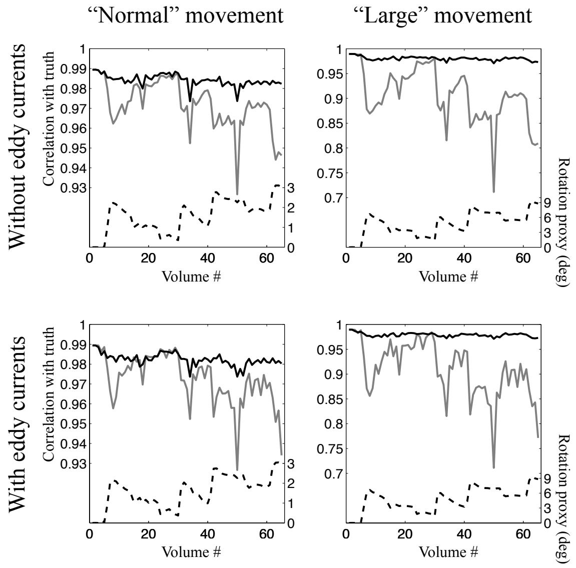


Figure S7: Results for slice 15 of simulations with volumetric movement, PE in the $A \rightarrow P$ direction and an SNR of 40

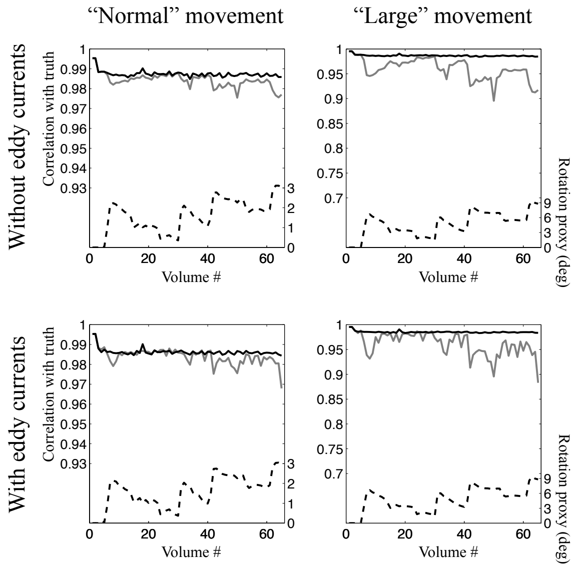


Figure S8: Results for slice 25 of simulations with volumetric movement, PE in the $A \rightarrow P$ direction and an SNR of 40

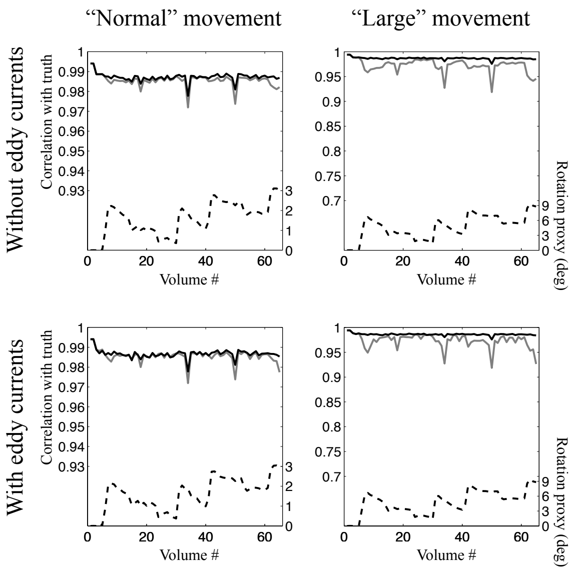


Figure S9: Results for slice 35 of simulations with volumetric movement, PE in the $A \to P$ direction and an SNR of 40

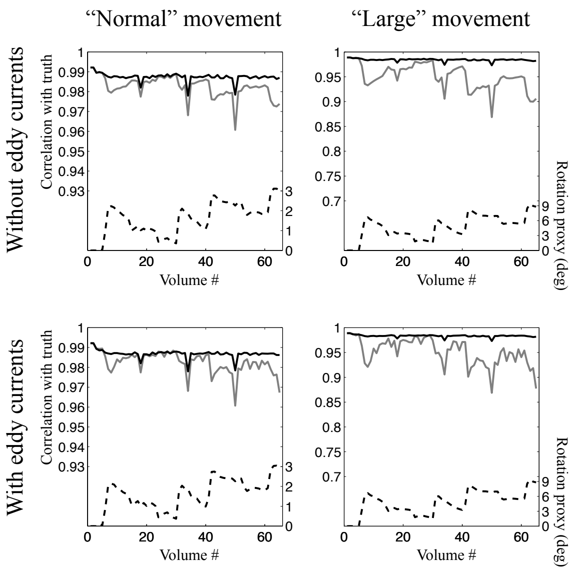


Figure S10: Results for the whole brain of simulations with volumetric movement, PE in the $A \to P$ direction and an SNR of 40

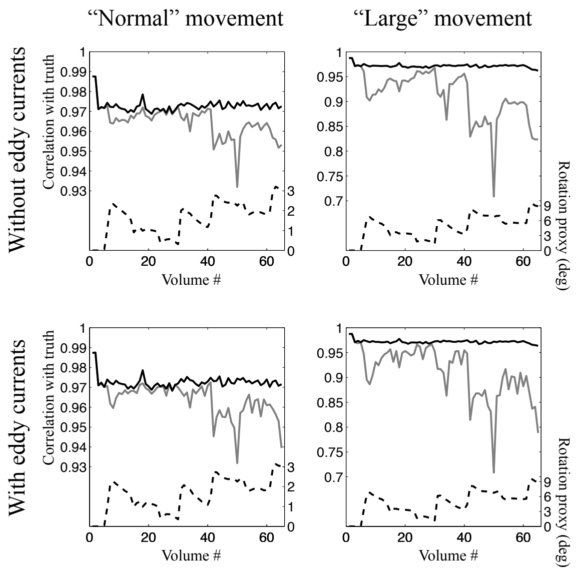


Figure S11: Results for slice 15 of simulations with volumetric movement, PE in the $P \rightarrow A$ direction and an SNR of 20

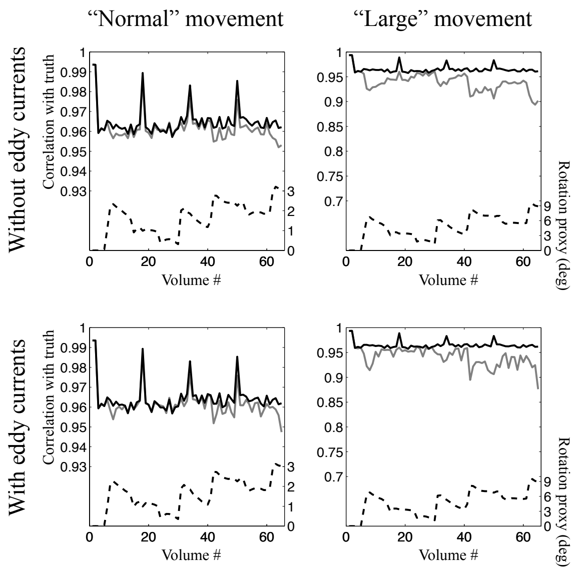


Figure S12: Results for slice 25 of simulations with volumetric movement, PE in the $P \rightarrow A$ direction and an SNR of 20

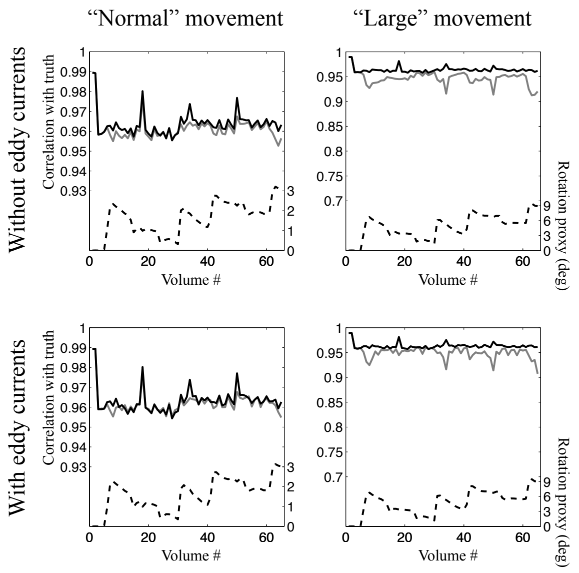


Figure S13: Results for slice 35 of simulations with volumetric movement, PE in the $P \to A$ direction and an SNR of 20

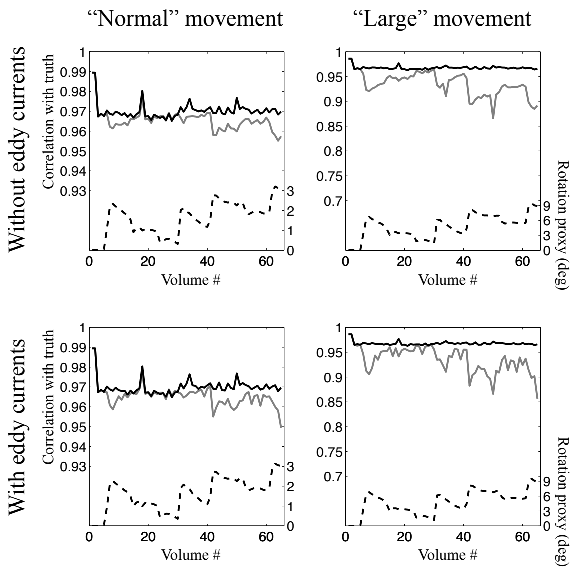


Figure S14: Results for the whole brain of simulations with volumetric movement, PE in the $P \to A$ direction and an SNR of 20

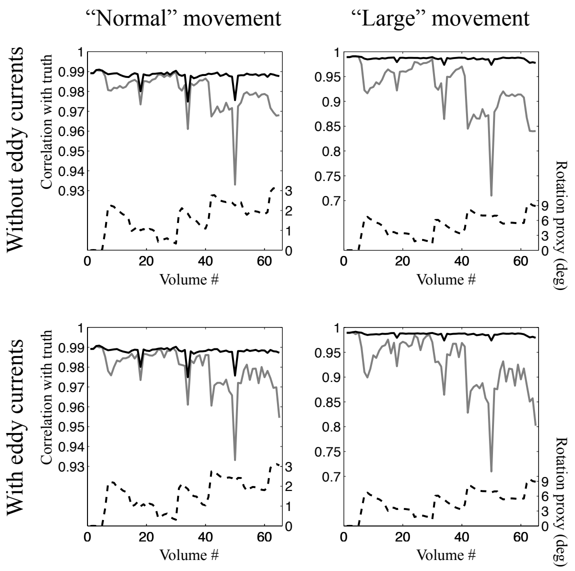


Figure S15: Results for slice 15 of simulations with volumetric movement, PE in the $P \to A$ direction and an SNR of 40

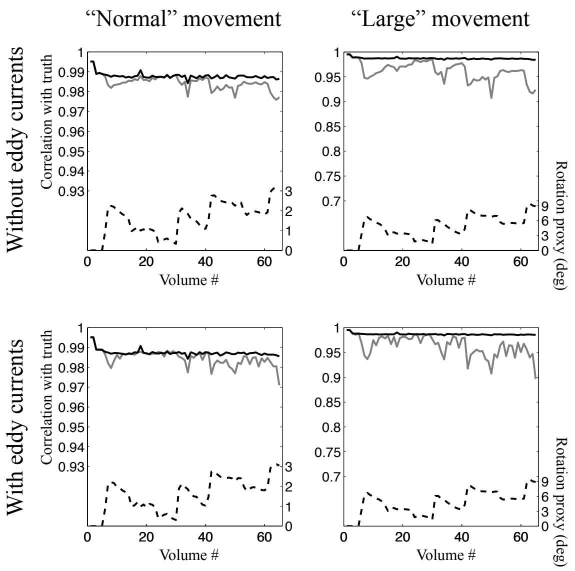


Figure S16: Results for slice 25 of simulations with volumetric movement, PE in the $P \to A$ direction and an SNR of 40

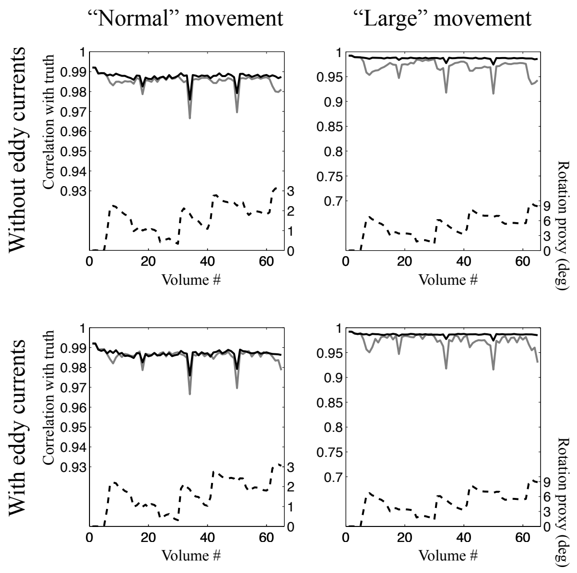


Figure S17: Results for slice 35 of simulations with volumetric movement, PE in the $P \rightarrow A$ direction and an SNR of 40

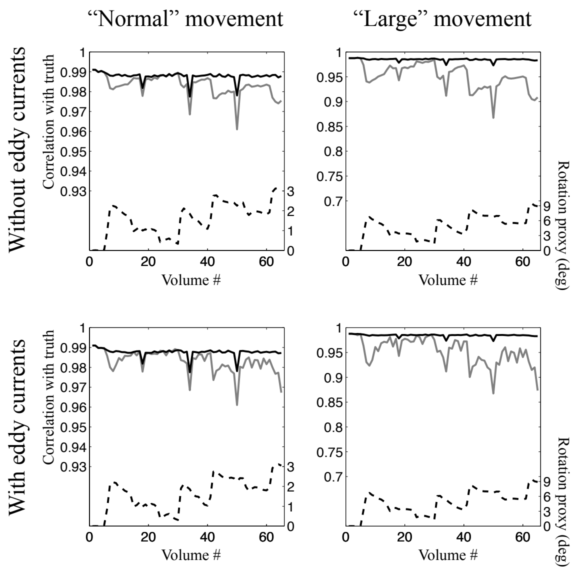


Figure S18: Results for the whole brain of simulations with volumetric movement, PE in the $P \rightarrow A$ direction and an SNR of 40

#### S.3.0.2 Simulations with intra-volume (slice-to-volume) movement.

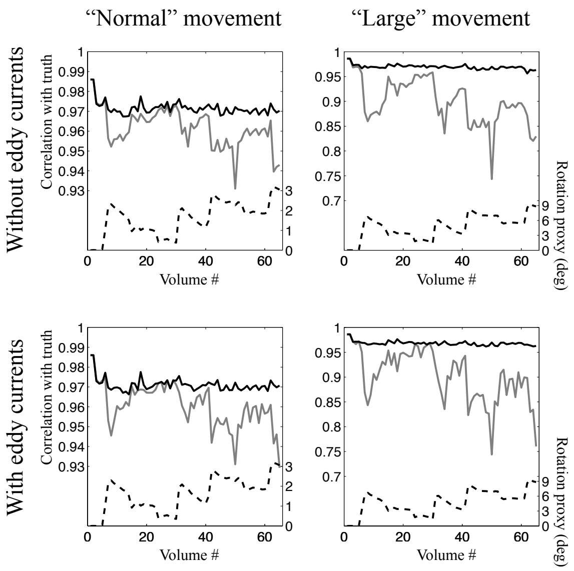


Figure S19: Results for slice 15 of simulations with slice-to-volume movement, PE in the $A \to P$ direction and an SNR of 20

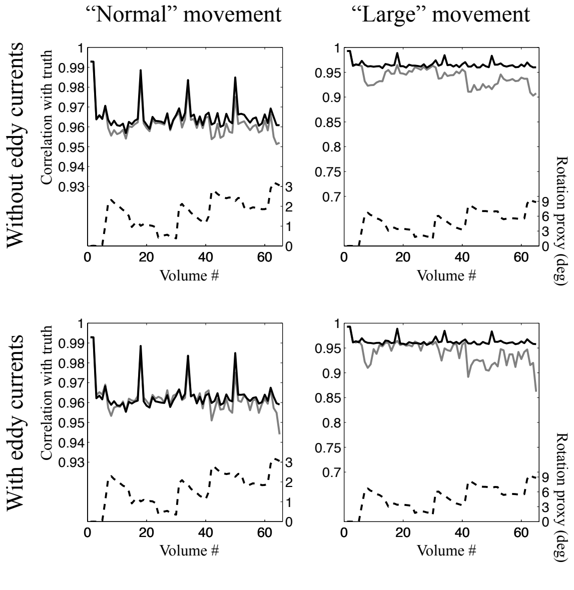


Figure S20: Results for slice 25 of simulations with slice-to-volume movement, PE in the $A \to P$ direction and an SNR of 20

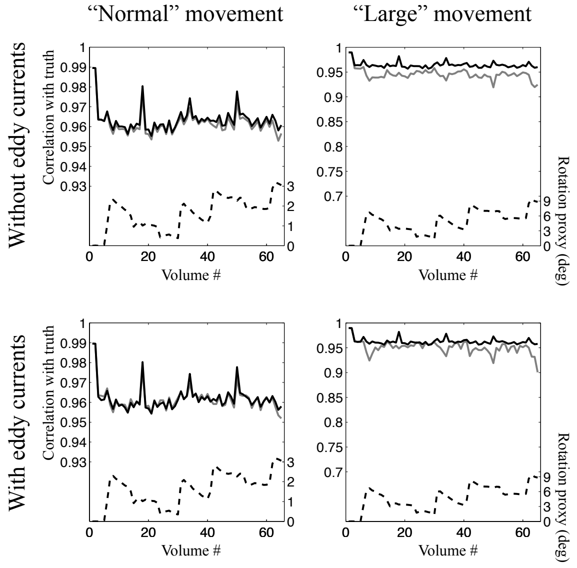


Figure S21: Results for slice 35 of simulations with slice-to-volume movement, PE in the $A \to P$ direction and an SNR of 20

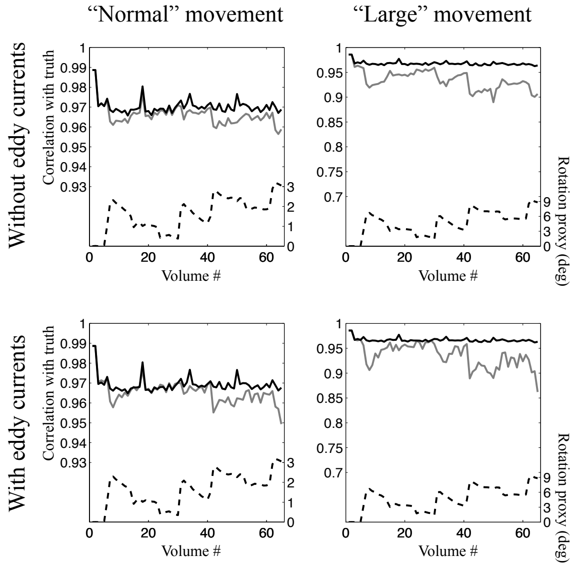


Figure S22: Results for the whole brain of simulations with slice-to-volume movement, PE in the $A \to P$ direction and an SNR of 20

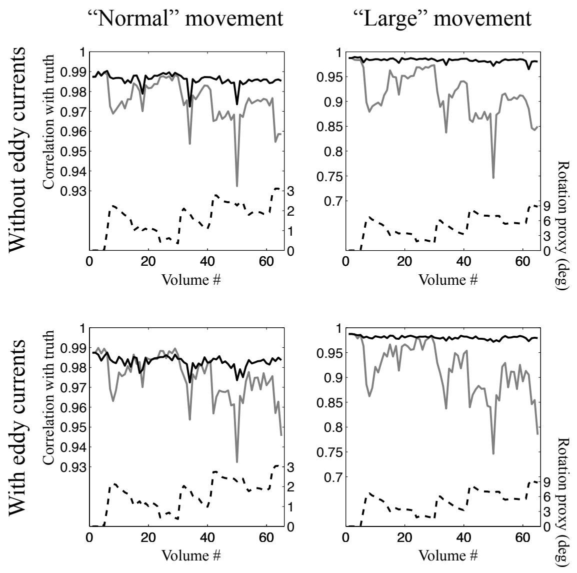


Figure S23: Results for slice 15 of simulations with slice-to-volume movement, PE in the $A \rightarrow P$ direction and an SNR of 40

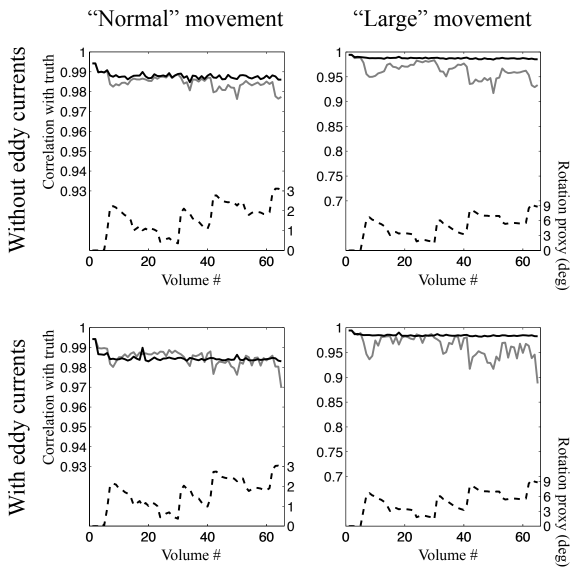


Figure S24: Results for slice 25 of simulations with slice-to-volume movement, PE in the $A \rightarrow P$ direction and an SNR of 40

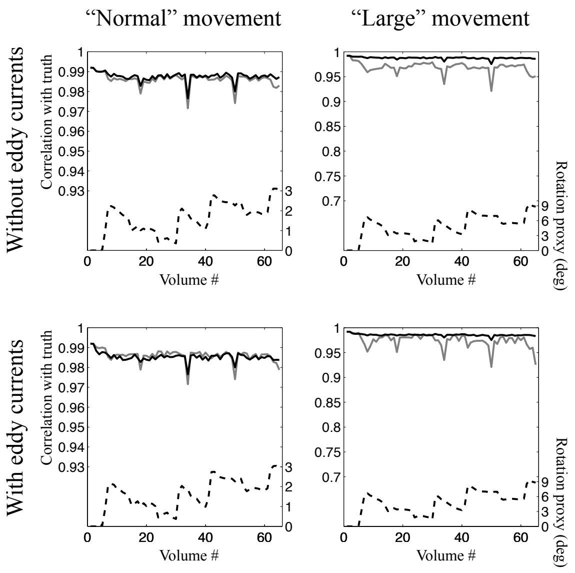


Figure S25: Results for slice 35 of simulations with slice-to-volume movement, PE in the $A \to P$ direction and an SNR of 40

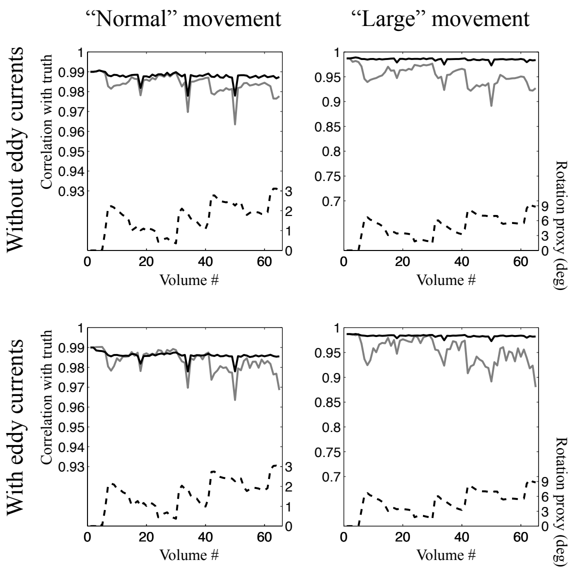


Figure S26: Results for the whole brain of simulations with slice-to-volume movement, PE in the $A \to P$ direction and an SNR of 40

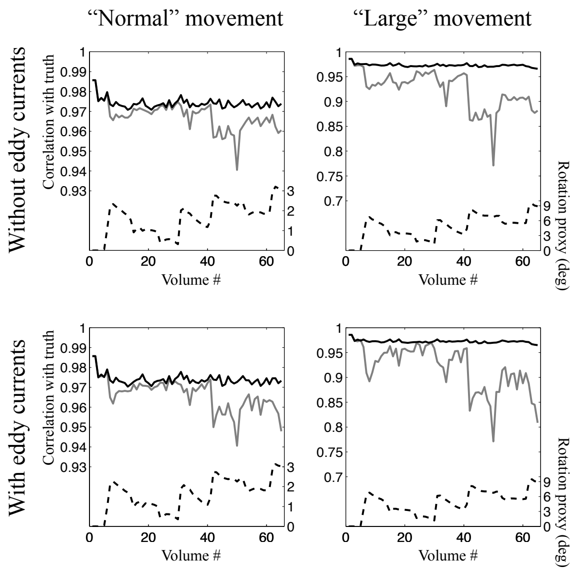


Figure S27: Results for slice 15 of simulations with slice-to-volume movement, PE in the $P \to A$ direction and an SNR of 20

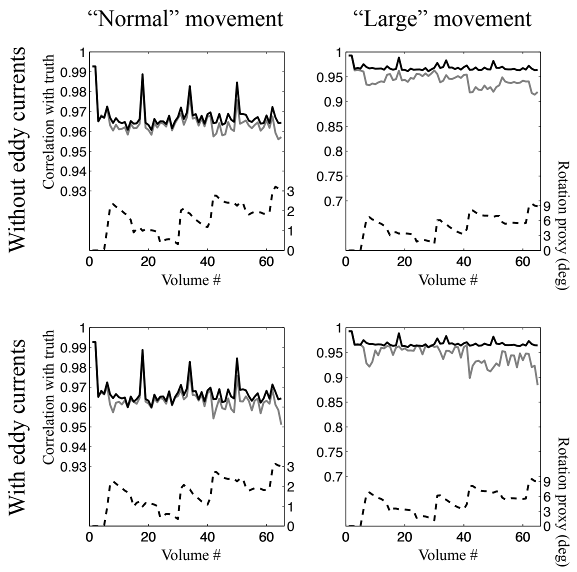


Figure S28: Results for slice 25 of simulations with slice-to-volume movement, PE in the $P \to A$ direction and an SNR of 20

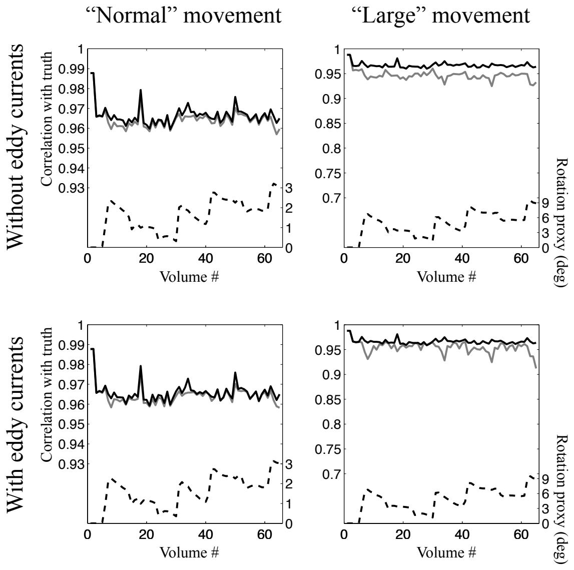


Figure S29: Results for slice 35 of simulations with slice-to-volume movement, PE in the $P \rightarrow A$ direction and an SNR of 20

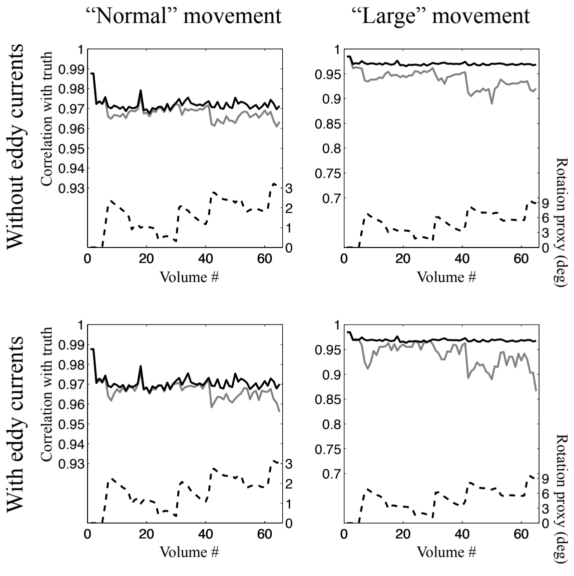


Figure S30: Results for the whole brain of simulations with slice-to-volume movement, PE in the $P \rightarrow A$ direction and an SNR of 20

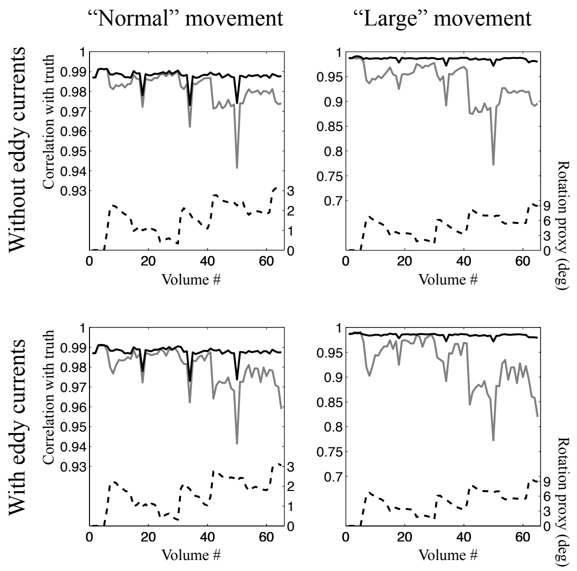


Figure S31: Results for slice 15 of simulations with slice-to-volume movement, PE in the $P \rightarrow A$ direction and an SNR of 40

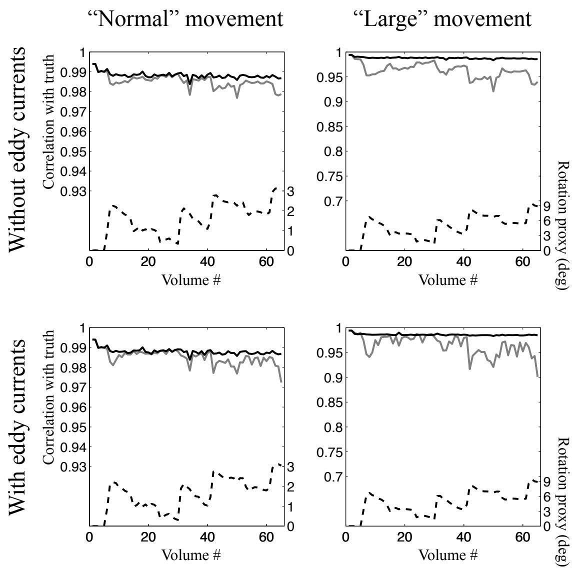


Figure S32: Results for slice 25 of simulations with slice-to-volume movement, PE in the $P \rightarrow A$ direction and an SNR of 40

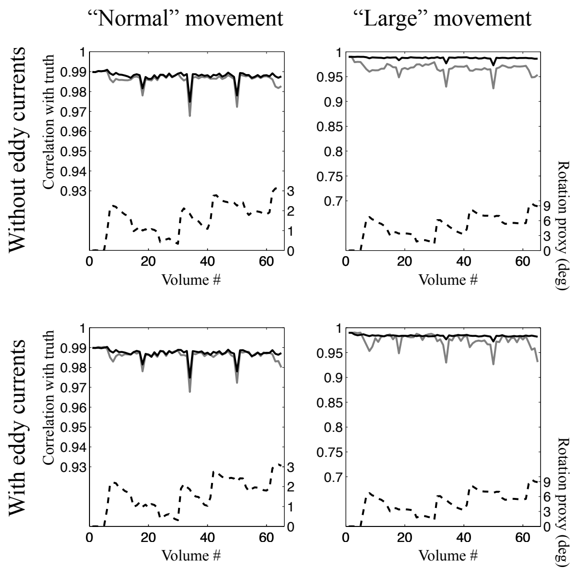


Figure S33: Results for slice 35 of simulations with slice-to-volume movement, PE in the $P \to A$ direction and an SNR of 40

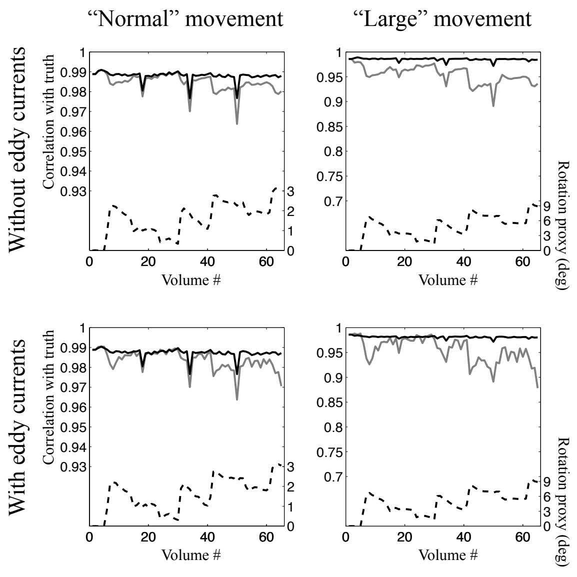


Figure S34: Results for the whole brain of simulations with slice-to-volume movement, PE in the $P \to A$ direction and an SNR of 40
